# The Effectiveness of Partnerships With Commercial Actors to Improve Food Environments: A Systematic Review

**DOI:** 10.1111/obr.13952

**Published:** 2025-06-29

**Authors:** Laurence Blanchard, Gemma Bridge, Julia Bidonde, Matt Egan, Harry Rutter, Mark Petticrew, Patricia J. Lucas, Monique Potvin Kent, Claire Bennet, Stephanie Ray, Cherry Law, Cécile Knai

**Affiliations:** ^1^ Faculty of Public Health Policy London School of Hygiene & Tropical Medicine London UK; ^2^ School of Earth and Environment University of Leeds Leeds UK; ^3^ York Business School York St John University York UK; ^4^ School of Rehabilitation Science, College of Medicine University of Saskatchewan Saskatoon Canada; ^5^ Department of Social & Policy Sciences University of Bath Bath UK; ^6^ SPECTRUM Consortium Edinburgh UK; ^7^ School for Policy Studies University of Bristol Bristol UK; ^8^ School of Epidemiology and Public Health University of Ottawa Ottawa Canada; ^9^ Public Health and Wellbeing Team Greenwich Borough London UK; ^10^ Department of Agri‐Food Economics and Marketing University of Reading Reading UK

**Keywords:** food environment, policy, public–private partnership, systematic review

## Abstract

Partnerships with commercial actors have been proposed as a policy approach to create healthier food environments. We conducted a systematic review to assess their effectiveness for improving food environments and population health at state, national, or international levels. We searched in 14 databases and two websites for real‐world evaluations published between 2010 and 2020. Study quality was appraised using a modified Newcastle–Ottawa Scale. Data were synthesized narratively by outcome (human, food environment, policy content, and implementation progress), considering their effect direction. Seventeen studies reporting on seven PPPs in four countries were included. Most studies (*n* = 14) involved food reformulation, especially salt reduction. Three focused on specific settings (the eating out‐of‐home sector, schools, and convenience stores). There was mixed evidence that partnerships make people buy fewer calories or more school meals (*n* = 3 studies) or reduce product sodium content (*n* = 6). Some positive effects were described in one uncontrolled study each for decreasing trans‐fatty acid intake and for making healthier options more available in school cafeterias, but these studies had important limitations. Five document analyses highlighted shortcomings in the partnerships, including their limited scope, failure to add value to ongoing actions, varying participation levels, and lack of implementation, monitoring, and reporting. Alternative policy approaches should be considered. This systematic review is registered on PROSPERO as CRD42020170963.

**Abbreviations**
AWASH: the Australian Division of World Action on Salt and Health
FHD: Food and Health Dialogue
HWC: Healthy Weight Commitment
NCD: noncommunicable disease
NGO: nongovernmental organization
NP: nonparticipants
NR: not reported
P: participants
PHA: Partnership for a Healthier America
PHRD: Public Health Responsibility Deal
PPP: public–private partnership
TFA: trans‐fatty acid

## 1 Introduction

Unhealthy diets, especially those high in sodium and low in fruit, whole grains, nuts, and seeds, are now one of the main risk factors for deaths and disability‐adjusted life years globally [1]. This is mainly due to the increased risk of cardiovascular diseases, type II diabetes, and some cancers [1]. Furthermore, high intakes of food and beverages high in fat, sugar, and/or salt (HFSS) are associated with obesity [2]. In 2022, 2.5 billion adults, 390 million children aged 5–19, and 37 million under‐5 children lived with overweight or obesity [2].

A leading driver for these dietary patterns is the nature of “food environments,” which according to the Food and Agriculture Organization (FAO) [3], encompass food availability, physical access (proximity), economic access (affordability), marketing of food products and health (including promotion and provision of information), and food quality and safety. Food environments have been extensively studied around the world, including the nutritional value of food and beverages offered in various settings; their physical access in a geographic area; their price and marketing strategies; and the nutritional information provided, for example, on labels or menus [4, 5]. Overall, studies show that many HFSS products are cheap and easily accessible, particularly in countries with higher income per capita and education levels, in lower income neighborhoods, in convenience stores and supermarkets, and in the vicinity of schools, and are highly promoted [3, 6, 7]. Such exposure is associated with a higher consumption of HFSS products and higher obesity rates [8, 9, 10].

It is now widely accepted that initiatives to promote healthy diets should aim at creating healthier food environments, rather than primarily targeting individual behavior change [11]. The importance of involving commercial actors in such initiatives is often assumed. Indeed, major global health commitments such as the Declaration of Alma Ata [12], the Bangkok Charter for Health Promotion in a Globalized World [13], and the UN Sustainable Development Goals [14], refer to multi‐stakeholder collaboration, alliances, partnerships, or other terms to refer to participation of commercial and other actors. The rationale is that commercial actors are major food system actors and that actions to address health problems should involve all key stakeholders [15, 16].

Commercial actors' participation in initiatives to improve the food environment can take the form of public–private partnerships (PPPs), joint ventures between at least one public actor (such as government) and at least one private actor (such as food producers, manufacturers, retailers, and/or service outlets), or partnerships between the public and voluntary sectors alone [17]. Initiatives can aim, for example, to reduce salt, sugar, or fat content of food and beverages; increase fruit and vegetable (FV) content; provide nutritional information; and/or limit the marketing of unhealthy products [18]. PPPs have been suggested to be cheaper, quicker, and more effective than legislation by harnessing private sector resources, efficiencies, reach, and expertise [15, 16]. Nevertheless, they have been criticized for not achieving public health objectives. The involvement of the food industry in public health policies has been particularly contested as a conflict of interest [16, 19].

To our knowledge, no systematic review has assessed the effectiveness of partnerships with the private sector (referred hereafter as “partnerships”) for improving food environments or population health. A systematic review by Parker et al. included PPPs for promoting health in general [20], and another by Harrison in 2024 focused on PPPs for preventing and managing obesity among children [21]. In both reviews, the analysis mainly focused on a high‐level description of the PPP's partners and topics rather than on their effects. Additionally, in the review by Harrison, the majority of interventions focused on weight management and education rather than on the food environment. The primary objective of our systematic review was to evaluate the evidence on effectiveness of partnerships with the private sector (at the state, national, and international levels) at improving food environments and population health. A secondary objective was to explore whether such partnerships are considered appropriate mechanisms for encouraging commercial actors to implement agreed changes, by assessing the policies' content and implementation progress.

## 2 Methods

This systematic review was conducted as part of a project evaluating the effectiveness, cost‐effectiveness, development, and implementation of regulatory and voluntary policies promoting healthy food environments at the state, national, and international levels, registered on PROSPERO (CRD42020170963). The project included evidence syntheses summarizing the evidence on different policy approaches. The detailed methods, including the eligibility criteria and literature searches, are provided in the project report [22]. A protocol was submitted to the funder (National Institute for Health and Care Research, England). Deviations include evaluating study quality using a modified Newcastle–Ottawa Scale instead of ROBINS‐I [23], not using the GRADE framework (see Section 4), and including all partnerships with commercial actors, not just PPPs. This systematic review is reported using the PRISMA 2020 checklist [24] (Table S1).

### 2.1 Literature Searches

As part of our larger project, we first conducted an evidence map, which served as a starting point for this systematic review [18]. We conducted searches in 14 international databases in November 2020 (ABI/INFORM Global, Campbell Collaboration, Cochrane Library, EconLit, Embase Classic + Embase, Epistemonikos, Medline, PsycINFO, Science Citation Index Expanded, Social Sciences Citation Index, Arts & Humanities Citation Index, Conference Proceedings Citation Index [Science], Conference Proceedings Citation Index [Social Science & Humanities], and Emerging Sources Citation Index). The search strategy included free text and controlled vocabulary structured around the concept of (regulatory OR PPP OR voluntary) AND policy AND diet. The search strategy in Medline was peer‐reviewed by a librarian using the Peer Review of Electronic Search Strategies statement [25] and is presented in Table S2. The NOURISHING database (https://policydatabase.wcrf.org/), the Global Food Research Program website (https://www.globalfoodresearchprogram.org/), and reference lists of evidence syntheses conducted within our project were screened for eligibility.

### 2.2 Eligibility Criteria

The evidence map included primary studies of “real‐world” regulatory and voluntary (including partnerships) policies targeting the general public and modifying food proximity, affordability, marketing, or information from anywhere in the world. Policies and evaluations needed to be conducted at the international, national, or state level. Evaluations were published between January 2010 and November 2020. There were no geographic or language restrictions. “Real world” referred to data being collected when the policy was adopted, implemented, or discussed in a public consultation. To be categorized as a partnership, policies needed to be explicitly labeled as such or needed to involve a collaboration (beyond simple consultation) between the private sector and either the public or voluntary sector. Records were screened independently by L.B., C.K., S.R., and C.L. using the EPPI‐Reviewer Web (EPPI‐Centre, University College London, UK).

For this systematic review, potential records were screened by two independent reviewers (L.B. and C.K.) against three additional criteria. Firstly, quantitative studies were included if they assessed partnerships' effectiveness on (a) human outcomes (e.g., food purchases, dietary intake, use of labels, and health) or (b) food environment outcomes (e.g., characteristics of food products, menus, physical places, or advertising). Secondly, secondary analyses of documents (“document analyses”) were included if they (a) employed a research methodology including data collection and analysis AND (b) evaluated policy documents, guidelines, reports, websites, emails, newsletters, media releases, or other sources produced by or for the partnership AND (c) assessed the design or content of the partnership (e.g., its objectives) and/or its implementation progress (e.g., the types of actions reported to be conducted to achieve the objectives). Analyses of media coverage of a partnership were excluded. As such, the systematic review included studies with human participants, food products, outlets, and documents (including websites and other digital formats). Thirdly, studies aggregating findings for partnerships and other policy approaches were excluded.

### 2.3 Data Extraction

Data were extracted in a standardized form in Word, including (a) partnerships' characteristics (countries, objective, products targeted, and actors and policy area using the World Cancer Research Fund NOURISHING framework [26]: N, labeling; O, public institutions; U, economic tools; R, advertising control; I, improving food supply, and S, retail and food services); (b) evaluation characteristics (study aim, study design, data collection dates, samples' characteristics; the smallest sample size of the first or last data collection was recorded; we categorized samples included in or targeted by a partnership as “participants” [P; e.g., customers at participating outlets or products from companies committed to the partnership] and others as “nonparticipants” [NP]); (c) results regarding human, food environment, policy content, and policy implementation progress outcomes, including any effect size and precision estimates, where applicable; (d) potential competing interests of study authors. Data were extracted by one reviewer (C.K. or G.B.) and checked by another (C.K., G.B., J.B., or L.B.).

### 2.4 Study Quality Appraisal

Study quality was assessed by outcome using a modified version of the Newcastle–Ottawa Scale for cross‐sectional studies [27]. The original tool and the modifications are presented in Table S2. The latter included guidance for studies assessing food environment outcomes and documents, as no tool currently exists for these. As in the original tool, seven methodological items were rated: (1) representativeness of sample, (2) sample size, (3) nonrespondents, (4) ascertainment/measurement of the exposure, (5) control of confounding factors, (6) outcome assessment, and (7) statistical test. Regarding modifications, firstly, partnership exposure could not be directly measured, and lack of implementation could be an evaluation outcome. To appraise “ascertainment of the exposure,” we assessed whether the lists of participating actors were outdated or changed during the evaluation period without being accounted for, using information in the studies and PPP documents. Secondly, we added an eighth item, “missing data,” to appraise handling methods. This was to compensate for the nonapplicability of “nonrespondents” on food environment outcomes and documents and was applied to all studies. Thirdly, identifying confounding factors in studies of food environment outcomes was challenging as typical confounders relate to human characteristics. Identified factors affected external validity (e.g., the time of the year or day of data collection) rather than internal validity. We gave a “low” quality rating to all studies not controlled for participating in the partnership (e.g., they evaluated only partnership participants or aggregated data on Ps and NPs). The remaining studies of food environment outcomes were rated as “moderate,” and those of human outcomes were appraised for their confounders. Lastly, the original tool used a scoring system, which Cochrane discourages [28]. We developed rating categories (low, moderate, high, or unclear) guided by the ROBINS‐I and ROB 2 tools [23, 29]. Studies with at least one “low” rating were classified as “low” quality. Studies rated “moderate” or “unclear” quality for two key items (4 and 7) could not be rated higher than these ratings. More than one “unclear” ratings resulted in an overall “unclear” classification unless an item was “low.”

For document analyses, we appraised sample representativeness by judging whether the literature search strategy comprehensively addressed the study aim by considering the nature and number of sources and keywords searched when applicable. For “sample size justification,” we assessed the variety of information sources used. “Statistical test” was replaced with “analytical methods” to include descriptive approaches. “Ascertainment of exposure” and “confounders” were deemed nonapplicable because the documents were about the policies themselves. Each item was assessed by one reviewer and checked by two others (J.B., G.B., and L.B.). Disagreements were resolved by consensus. The modifications were tested iteratively by L.B., J.B., and G.B. until agreement was reached.

### 2.5 Data Synthesis

Due to high heterogeneity in the interventions, outcomes, and effect measures assessed, data from studies of human and food environment outcomes were synthesized narratively by outcome category, considering the number of studies, sample size, study quality, and control for participating in a partnership. Studies collecting data at a minimum of two points in time were also given a direction of effect for each outcome category. At the review level, an overall direction of effect was then determined for each outcome category. The latter could be (a) positive, when at least 70% of study‐level outcomes were positive [30, 31]. Positive evidence from a single study or fewer than 300 participants (inspired by Hilton Boon and Thomson [30]) was described as “some” positive evidence; (b) mixed, when the majority was not statistically significant or was contradictory; and (c) negative, when the majority showed worsened effects. The effect directions were presented by partnership, quality (from highest to unclear), and publication date (most to least recent) in an effect direction plot, which visually represents a summary of findings for multiple heterogeneous and nonstandardized outcomes [32]. Findings from studies not controlled for partnership participation were presented separately in both the tabulation and the text.

Data from the document analyses were synthesized by themes relating to the partnerships' characteristics, employing a thematic synthesis approach.

Heterogeneity was explored by effect direction, considering the partnership, country, study design, policy area, study quality, evaluation period, and potential competing interests.

## 3 Results

**FIGURE 1:**
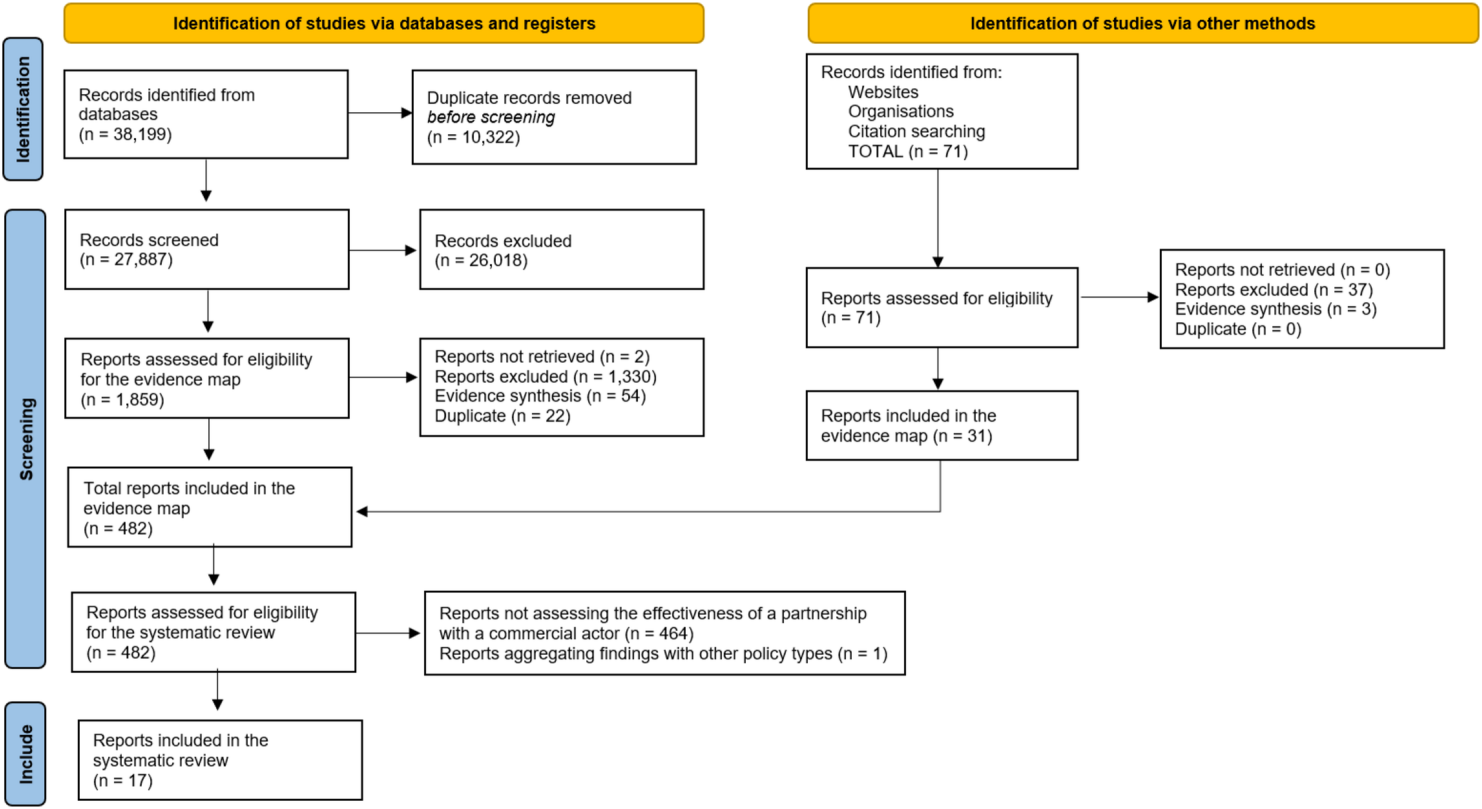
Flowchart representing the selection process. PRISMA template from [24]. For more information, visit http://www.prisma‐statement.org/.

Figure 1 summarizes the study selection process for the evidence map (detailed in [22]) and this systematic review. From the bibliographic databases, 38,199 records were identified, of which 27,887 remained after removing duplicates. Of these, 1859 met the criteria and had their full text screened. Another 71 additional full texts were identified on the websites and reference lists, leading to 482 publications being included in the evidence map. From these, 18 studies assessed the effectiveness of a partnership with the private sector. One was excluded for not providing data on partnerships alone [33], leaving 17 studies in the systematic review.

### 3.1 Characteristics of Partnerships

The 17 studies report on seven partnerships implemented in four countries between 2007 and 2016. The partnerships are described in Table 1. Half (*n* = 8) of studies were about the Food and Health Dialogue (FHD), a PPP implemented in 2009 by the Australian government with the food industry and health advocacy groups. It aimed to reformulate multiple food and beverage categories (i.e., to modify their composition) by setting up nutritional targets, as well as to standardize portion sizes and promote consumer education [34]. The FHD was replaced in 2018 by the Healthy Food Partnership. One study evaluated the Drop the Salt! campaign, implemented in 2007 by the Australian Division of World Action on Salt and Health (AWASH, not‐for‐profit). AWASH collaborated with the Australian Food and Grocery Council (industry association) and food companies to reduce average population salt intake by 25% by 2025 [35, 36]. One study assessed both the FHD and Project Target 450 [37]. The latter was led in 2007 by the New Zealand Heart Foundation (not‐for‐profit) in collaboration with major bread manufacturers to reduce sodium content in breads below 450 mg/100 g.

**TABLE 1:** Characteristics of the partnerships (*n* = 7).

Name	Policy areas assessed	Objective	Impl. (I) and eval. (E) years	Partnership lead and partners	Related studies; food items evaluated overall
**Australia**					
AWASH Drop the Salt! Campaign	I: Food reformulation (sodium)	To reduce average population salt intake by 25% by 2025 [44] Products targeted: The 2011 targets covered a wide range including bread and bakery, cereals, convenience, fast food, FV, meat, sauces, and snacks [35]	I: 2007 E: 2007–2011	Lead: AWASH (NGO) Partners: Australian Food and Grocery Council and individual food companies	1 effectiveness study: Ready meals [45]
Food and Health Dialogue (FHD)	I: Food reformulation (a wide range)	To decrease the saturated fat, added sugar, sodium, and energy content and increase the FV, fiber, and wholegrain content of foods [34] Products targeted: A wide range including grain, soups, snacks, meat, fish, dairy, FV, and nonalcoholic beverages [36]	I: 2009 E: 2010–2017	Lead: Australian government Partners: Food industry and health advocacy groups	5 effectiveness studies: Bread, pasta sauce, processed meats, and soups [34, 37, 41, 46, 47] 3 document analyses: All food categories [36, 48, 49]
**New Zealand**					
Project Target 450	I: Food reformulation (sodium)	Project Target 450: To engage the industry in reducing sodium content in low‐cost, high‐volume bread products to ≤ 450 mg/100 g [ 50 ] Products targeted: Bread	I: 2007 E: 2007–10	Lead: New Zealand Heart Foundation (NGO) Partners: Main bread manufacturers	1 effectiveness study: Bread [37]
**United Kingdom**					
Public Health Responsibility Deal (PHRD), England, UK	I: Food reformulation (fat, sodium, calories, FV) N: Labeling (out‐of‐home sector and front‐of‐packs)	8 food pledges relating to out‐of‐home calorie labeling; front‐of‐pack labeling; salt, calorie, saturated fat, and TFA reduction; portion sizes; and FV Products targeted: All (no specific category)	I: 2011 E: 2000–18	Lead: English Department of Health (DOH) Partners: Commercial actors (majority) and NGOs	2 effectiveness studies: TFAs, calorie displays [42, 51] 2 document analyses: All products, 7 pledges (not salt) [43, 52]
**USA**					
Choose Healthy Now	S: Retail and food service sectors (convenience stores)	To improve the healthfulness of snacks and drinks in convenience stores and snack shops using product placement, in‐store signage, coupons, discounts, tastings, and healthy offerings Products targeted: A wide range including grain, soups, snacks, meat, fish, dairy, FV, and nonalcoholic beverages [53]	I: 2016 E: 2018	Lead: Hawai'i State DOH Partners: 2 convenience store chains (Aloha Island Mart and 7‐Eleven Hawai'i)	1 effectiveness study: All products, emphasis on snacks, fruits, and nonalcoholic beverages [39]
Healthy Weight Commitment (HWC)	I: Food reformulation (calorie reduction)	To collectively sell 1 trillion fewer calories in the United States by 2012 compared to 2007 and 1.5 trillion fewer by 2015, in order to help reduce obesity (especially in children) [38] Products targeted: All (no specific category)	I: 2009 E: 2000–12	Lead: HWC Foundation (16 major food manufacturers^a^) Partners: Partnership for America, White House, and Robert Wood Johnson Foundation [54]	2 effectiveness studies: All products [55, 56]
Strong4Life School Nutrition Program	O‐Schools	To increase school meal participation and consumption of healthier foods in Georgia school cafeterias by better equipping managers and staff members with skills and resources to make positive and visible changes in the cafeteria Products targeted: All, emphasis on FV	I: 2014 E: 2015	Lead: Children's Healthcare of Atlanta and schools Partners: Georgia Shape (Governor of Georgia) and schools	1 effectiveness study: National School Lunch Program [40]

Abbreviations: AWASH = the Australian Division of World Action on Salt and Health; DOH = Department of Health (government); FHD = Food and Health Dialogue; FV = fruit and vegetables; HWC = Healthy Weight Commitment; NGO = nongovernmental organization; PHRD = Public Health Responsibility Deal; TFA = trans‐fatty acid.

^a^ Bumble Bee Foods LLC; Campbell Soup Company; Conagra Foods; General Mills Inc.; Kellogg Company; Kraft Foods Inc. (now consisting of the Kraft Heinz Company and Mondelēz International); Mars Incorporated; McCormick & Company Inc.; Nestlé USA; PepsiCo Inc.; Post Foods/Ralston Foods LLC; Hillshire Brands (part of Tyson Foods Inc. and was previously the Sara Lee Corporation); The Coca‐Cola Company; The Hershey Company, The J.M. Smucker Company; and Unilever.

Four studies evaluated the Public Health Responsibility Deal (PHRD), a PPP led by the then Department of Health in England, UK, between 2011 and 2015, along with commercial actors (including food and alcohol industry representatives) and some nongovernmental organizations. Partners could sign up to pledges, including eight relating to food (about out‐of‐home calorie labeling, front‐of‐pack labeling, FV, salt in the catering trade as well as salt, saturated fat, trans fat and calorie reduction).

Another four studies investigated three PPPs in the United States. Two evaluated the Healthy Weight Commitment (HWC), which was initiated in October 2009 by a group of 16 leading American food manufacturers. It was formalized through an agreement in May 2010 with Partnership for a Healthier America (PHA; a not‐for‐profit created as part of First Lady Michelle Obama's *Let Us Move!* campaign and in which Mrs Obama was honorary chair) and announced by the White House [38]. The companies participating in the HWC pledged to sell all together one trillion fewer calories in the United States by 2012 compared to 2007 and 1.5 trillion fewer by 2015. One study focused on the Hawai'i Department of Health (DOH)–led Choose Healthy Now [39]. Initiated in 2016, the program encouraged two convenience store chains to promote healthy options using education and structural interventions. The last study assessed the Strong4Life School Nutrition Program (named hereafter “Strong4Life”), developed in 2014 by Children's Healthcare of Atlanta (a large pediatric clinical care provider) in partnership with Georgia Shape (the Georgia governor's childhood obesity initiative) [40]. It aimed to increase school meal participation and healthy food consumption in cafeterias by making healthier options more attractive, visible, convenient, and affordable in school cafeterias.

Overall, five partnerships represented in 15 studies involved food reformulation, mainly to reduce sodium content, whereas three focused on specific settings (the eating out‐of‐home sector, schools, and convenience stores). Five partnerships involved the public sector (i.e., they were PPPs: Choose Healthy Now, FHD, HWC, PHRD, and Strong4Life). The private sector contributed to the formulation of the scope and targets of all PPPs but Choose Healthy Now [17, 38, 39, 40], with the food industry said to have had substantial institutional power in some of them [17]. AWASH and Project Target 450 were partnerships with not‐for‐profit organizations relating to salt and were led and developed by the latter. Participation by commercial partners was highly variable. On the one hand, the FHD included 80% and 95% of market shares of breads and processed meats, respectively [41]. On the other hand, only 16 large eating‐out chains out of the 104 assessed by Robinson et al. had signed up to the PHRD calorie‐labeling pledge [42], and only 11 organizations out of 90 committed to fully removing trans‐fatty acids (TFAs) [43].

### 3.2 Characteristics of Included Studies

The study characteristics are described in Table 2 and detailed in Table S2. Two studies were cross‐sectional, nine were repeat cross‐sectional (four pre–post and six post–post, including one study that included both designs), one was longitudinal, and five were document analyses. Five studies assessed human outcomes (purchases or sales and TFA intake), eight assessed food environment characteristics (product sodium content, school cafeterias, and calorie‐labeling displays), and the five document analyses examined the content and implementation of the FHD and PHRD. Three studies of human and food environment outcomes evaluated the partnerships 1 year or less after implementation (including Dunford et al. for the FHD), five after 2–4 years (including Dunford et al. for Project Target 450), and five after 5–8 years. The sample size of “intervention/partnership” groups (except in document analyses) ranged from 16 restaurant chains, 59–181 food items, and 162 adults to over 60,000 households. Three and two document analyses assessed the partnerships after 2–4 years of implementation and after 5–8 years, respectively.

**TABLE 2:** Direction of effects by partnership, human, and food environment outcomes (*n* = 12).

Study details			Human outcomes		Food environment outcomes		
Author (year)	Study design; *N* years since implementation^a^ (study quality)	Sample size of participant (P) /intervention group^b^ (% of sample)	Sales and purchases	Dietary intake	Sodium reduction in food items	% products meeting sodium targets	Healthy foods availability and access
**Australia—AWASH Drop the Salt! (*n* = 1)**							
Christoforou et al. [45]	RCS post–post, 4 years, (−)^c^	107 ready meals (P and NP)			◀▶ ready meals	◀▶ ready meals	
**Australia—Food and Health Dialogue (FHD) (*n* = 5)**							
Trevena et al. [41]	RCS post–post, 4 years, (+)	145 (84%) breads, 83 (92%) processed meats, 86 (70%) breakfast cereals			◀▶ (bread and meat ◀▶, cereals ▲)	◀▶ (bread ▲, meat ◀▶)	
Sparks et al. [46]	RCS post–post, 8 years, (−)	181 (43%) processed meats			▲ meat		
Levi et al. [34]	RCS post–post, 5 years, (−)	59 (87%) dry soups, 124 (66%) wet soups			◀▶ soups (dry ▲, wet ◀▶)	◀▶ soups (dry ▲, wet ◀▶)	
Trevena et al. [47]	RCS post–post, 2 years, (−)^c^	124 pasta sauces in total (P and NP)			◀▶ pasta sauce	◀▶ pasta sauce	
Dunford et al. [37]	RCS pre–post, 1 year, (−)^c^	94 breads in total (P and NP)			◀▶ bread	▲ bread	
**New Zealand—Project Target 450 (*n* = 1)**							
Dunford et al. [37]	RCS pre–post, 3 years, (−)^c^	63 breads in total (P and NP)			▲ bread	▲ bread	
**UK—Public Health Responsibility Deal (PHRD) (*n* = 1)**							
Hutchinson et al. [51]	RCS pre–post, 1 year, (−)^c^	848 adults in total (P and NP)		▲ TFA			
Robinson et al. [42]: controlled CS with a single data collection—No effect direction allocated							
**USA—Healthy Weight Commitment (HWC) (*n* = 2)**							
Ng et al. [56]	Longitudinal pre–post, 5 years, (+)	Sales data from the 16 Ps linked with ≥ 60,000 households, compared with NPs	▲ calories sold				
Ng and Popkin [55]	RCS pre–post, 5 years, (+)	Purchases from 61,126 households from the 16 Ps compared with NPs	◀▶ calories purchased				
**USA—Choose Healthy Now (*n* = 1)**							
Beckelman et al. [39]: uncontrolled (P only) CS with a single data collection—No effect direction allocated							
**USA—Strong4Life School Nutrition Program (*n* = 1)**							
Rajbhandari‐Thapa et al. [40]	RCS pre–post, 1 month, (−)	80 schools (100% but controlled for attending training (50%–50%))	◀▶ school meals				
Rajbhandari‐Thapa et al. [40]	RCS post–post, 3 months, (−)^c^	325 staff and managers (100%)					▲ (self‐reported)
**Overall direction of effect**			◀▶	▲	◀▶	◀▶	▲

*Note:* Study quality: (−) = low; (+) = moderate, (++) = high; (?) = unclear. Effect direction: upward ▲ = positive; downward ▼ = negative/worsened; sideways ◀▶ = no change/mixed/conflicting. Sample size: large ▲ = > 300; medium ▲ = 50–300; small ▲ = < 50.

Abbreviations: AWASH = Australian Division of World Action on Salt and Health; CS = cross‐sectional; NP = nonparticipant; P = participant; RCS = repeat cross‐sectional.

^a^ *N* years between policy implementation and last data collection.

^b^ Smallest sample assessed.

^c^ Not controlled for partnership participation.

### 3.3 Quality Appraisal

Two‐thirds of studies of human and food environment outcomes (*n* = 8) were rated as low quality overall, three as moderate, one as unclear, and none as high (Tables S4 and S5). The main reason for low quality ratings was the absence of a counterfactual for partnership participation (*n* = 6, i.e., half of studies). Information on nonresponders in studies of human outcomes and justifications for sample size in studies of both humans and food environments were mostly inappropriate or unclear. Only six studies reported information on missing data and handling methods. Five studies did not mention whether data were collected and verified by two people or more independently. Regarding the document analyses, three were rated as high quality and two as low (Table S6). The study by Elliot et al. searched a limited number of information sources, especially when compared with Jones et al., who repeated the study (with some differences) 2 years later. Three document analyses did not provide information relating to missing data, and two did not provide information on whether data were collected and verified by two people or more independently. The analytical methods employed were mostly appropriate.

### 3.4 Human Outcomes

Five studies on four PPPs assessed human outcomes, including purchases or sales relating to three American PPP (*n* = 4) and TFA intake in the United Kingdom (*n* = 1) (Table 2, with details in Table S7). One of the studies on purchases and sales was cross‐sectional and so was not given an effect direction. Effects from the remaining three studies were mixed overall (*n* = over 60,000 households, studies of low, and moderate quality), including from a study that included only partnership participants but was controlled for attending a partnership's training. The first two studies, both led by Ng et al., used sales and household purchase data to evaluate the HWC. A pre–post cross‐sectional study (*n* = over 60,000 households, low quality) found that calories sold from participating companies between 2007 and 2012 decreased by over 10%, representing 6.4 trillion calories, thus six times more than the PPP's objective [44]. This corresponded to an average of −78 vs. −11 kcal/capita/day from nonparticipating companies. Then, a pre–post longitudinal study (*n* = 61,126 households) covering the years 2000–2012 evaluated calorie purchases using a model adjusted for consumer demands and economic factors [45]. That study showed that calorie purchases from participating brands remained higher than projections calculated using pre‐pledge data (2000–2008), whereas calorie purchases from nonparticipating brands decreased more than projected. It is worth noting that 2008 was used as the cutoff date rather than 2009 (date of launch by the industry) or 2010 (date of partnership with PHA).

Additionally, a pre–post controlled cross‐sectional study by Rajbhandari‐Thapa et al. (low quality) showed no evidence that a 90‐min in‐person staff training for the Strong4Life intervention in Georgia, USA (*n* = 40 schools), in addition to taking part in Strong4Life (*n* = 40 schools), increased students' participation in the National School Meal Program 1 month after the training, compared to 12 months before when the program started (*p* = 0.36) [40]. Lastly, Beckelman et al. conducted an uncontrolled cross‐sectional study (low quality, no effect direction allocated) to evaluate Hawai'i DOH's Choose Healthy Now program in participating convenience store chains 2 years after implementation [39]. The quarter of adults surveyed in stores (total *n* = 162) reported having bought at least one food or beverage that met the program's nutrition criteria. The authors (who included DOH staff) considered the initiative as a mutual benefit for the government and convenience stores.

Regarding dietary intake, there is some positive evidence from a pre–post cross‐sectional study by Hutchinson et al. (low quality) that the English PHRD reduced TFA intake in the United Kingdom. The authors analyzed National Diet and Nutrition Surveys, but the study was not controlled for partnership participation. The proportion of adults exceeding the World Health Organization TFA intake limit fell from 57% before the PHRD (2000–2011, *n* = 1724) to 2.5% after (2010–2012, *n* = 848) [46]. Higher consumption was associated pre‐PHRD with lower income and education attainment and greater levels of long‐term illness/disability, while post‐PHRD results were mixed. Data post‐PHRD were collected from 1 to 12 months after the PPP launch [47], which questions the attribution of success to the PPP, especially given the magnitude of improvement observed.

### 3.5 Food Environment Outcomes

Eight studies assessed the effect of a partnership on food environment outcomes, including product sodium content (*n* = 6), proportion of products meeting sodium targets (*n* = 4), changes to school food cafeterias (*n* = 1), and presence of calorie information in the eating out‐of‐home sector (*n* = 1).

The six studies that assessed sodium content were conducted in Australia and New Zealand. Three were controlled for partnership participation. All evaluated the FHD (implemented in 2009). Evidence was mixed overall (*n* = 678 participating products at one point in time, studies of low and moderate quality). Trevena et al. (moderate quality) found that mean sodium content decreased in bread (*n* = 145 participating products), processed meat (*n* = 83), and breakfast cereals (*n* = 86) between 2010 and 2013, but it only reduced significantly more in participating products compared to nonparticipating products for breakfast cereals (*p* = 0.005) [41]. Only 7–13 nonparticipating processed meats were included. By contrast, in the Sparks et al. study (low quality), median sodium levels in participating processed meats reduced by 11% (*n* = 181, *p* < 0.001) between 2010 and 2017 vs. no change in nonparticipating processed meats (*n* = 238) [48]. Median sodium content in nonparticipating products remained lower (717 mg/100 g in 2017 vs. 898 mg/100 g in participants), highlighting room for further improvement. Levi et al. (low quality) recorded significant sodium reductions between 2011 and 2014 in dry soups from both participating (*n* = 59) and nonparticipating products (*n* = 9; no effect estimates reported), but not wet soups (*n* = 124 Ps, 65 NPs) [34].

Three additional studies measured mean sodium content over time but analyzed products without distinguishing those participating in the partnership from those that do not. Evidence was mixed again (*n* = 388 products at one point in time, low quality). A different study by Trevena et al. (low quality) on the FHD noted no significant change in pasta sauces (*p* = 0.016) between 2008 (*n* = 124 products) and 2011 (*n* = 187), including when analyzing ambient and fresh sauces separately [49]. Dunford et al. (low quality) recorded no change in breads under the FHD between 2007 (*n* = 94, mean = 434 mg/100 g) and 2010 (*n* = 99, mean = 435 mg/100 g) [37]. However, in New Zealand, they found a significant 7% decrease in breads under Project Target 450, from 469 mg/100 g [453.690–484.310] in 2007 (*n* = 63) to 435 mg/100 g [422–447] in 2010 (*n* = 68) (95% CIs calculated using mean and standard deviation values provided in the article) [37]. Considering that the FHD manufacturers of pasta sauces and breads represented 85% and 80% of market shares, respectively [41, 49], the lack of change observed is likely to be due to lack of change in participating brands. Christoforou et al. (low quality) reported similar mean sodium levels in ready meals between 2008 (279 mg, *n* = 107) and 2011 (277 mg, *n* = 265) under Drop the Salt!, although sodium targets were introduced only in 2011 [50].

Five of the six studies above also calculated the proportion of products meeting sodium targets. Two were controlled for partnership participation and showed mixed evidence (*n* = 411 products at one point in time, low and moderate quality). Trevena et al. (moderate quality) found that a higher proportion of FHD breads (*n* = 145) met the target between 2010 and 2013 compared to nonparticipating breads (*n* = 27, *p* < 0.02) and found no significant difference for processed meats (*n* = 83 Ps, *n* = 7 NPs, *p* = 0.14) [41]. In the Levi et al. study (low quality), a greater proportion of FHD dry soups met the target in 2014 compared with 2010 (*p* < 0.0001), whereas no difference was detected for nonparticipating dry soups and both groups of wet/condensed soups [34]. Evidence was also mixed for three studies that analyzed without accounting for partnership participation (*n* = 388 products at one point in time, low quality). Trevena et al. (low quality) reported no significant change in pasta sauces under the FHD between 2008 (*n* = 124) and 2011 (*n* = 187, *p* = 0.16), including for ambient and fresh sauces [49]. In contrast to the first study by Trevena et al., the Dunford et al. study (low quality) found that the proportion of breads meeting the FHD target increased from 29% in 2007 (*n* = 94) to 50% in 2010 (*n* = 99) and from 49% for Project Target 450 in 2007 (*n* = 63) to 90% in 2010 (*n* = 68) [37]. In the Christoforou et al. (low quality) study, the proportion of ready meals meeting the targets slightly decreased between 2008 (59%, *n* = 107) and 2011 (57%, *n* = 265), although targets were introduced in 2011 [50].

There is some positive evidence from an uncontrolled post–post cross‐sectional study by Rajbhandari‐Thapa et al. (low quality) that staff made healthier food more available, convenient, and visible 3 months after attending a Strong4Life training, although data were self‐reported [40]. For example, 96% of participants indicated making healthy options available in at least two locations on each service line compared with 84% prior to training (*p* < 0.001). Lastly, Robinson et al. (unclear quality) evaluated the implementation of calorie labeling in 16 PHRD signatory eating‐out chains and 88 nonsignatory [42]. While the majority of chains that provided calorie information had committed to the pledge (12 vs. 6), none met all PHRD recommendations. No effect direction was allocated due to the study's cross‐sectional design.

### 3.6 Policy Content and Implementation

Five document analyses assessed the content or implementation progress of a partnership: three on the FHD and two on the PHRD. All were critical of the PPPs by reporting limitations relating to their design, implementation, participation, and/or monitoring.

Four document analyses evaluated the partnerships' design. Two highlighted the limited scope of the FHD. As part of the FHD, the Reformulation Working Group had identified priority food categories and organized industry roundtables to develop targets and action plans for these categories. Targets could be developed for up to eight “action areas” (sodium, saturated fat, added sugar, energy, fiber, whole grains, FV content, and portion size) depending on the food category. Elliot et al. (study rated low quality) examined the targets established in the first 4 years (2009–2013) using information from the FHD website, media releases, and newsletters [51]. They found that only 11 targets had been developed out of 124 possibilities. A second evaluation 2 years later (low quality) by Jones et al. included three additional food categories [36]. Only 12 out of 137 potential targets had been developed (the new one being about sodium in cheese). There were still no targets for added sugar, energy, fiber, whole grains, FV, and ready meals, and there were very few for saturated fat and portion size [36].

Two document analyses evaluated the “added value” of the PHRD's objectives. By analyzing 2015 progress reports, Knai et al. (high quality) estimated that TFA removal had already been completed or was ongoing at the PHRD launch for 91% of the 90 participating organizations [43]. Another study by the same authors (high quality) analyzed the progress reports from 2012 to 2014 from the 253 signatory organizations for six of the eight food pledges (excluding TFAs and salt) [52]. They reported an overall lack of “additionality” of the PHRD since most of reported actions were clearly (37%) or possibly (37%) already underway. For both the FHD and PHRD, an emphasis was noted on education, information provision, and awareness raising despite evidence showing that these interventions tend to be less effective than structural interventions [52, 53].

Regarding implementation, the three document analyses on the FHD highlighted a lack of action towards the objectives. Elliot et al. found that no action had been reported to meet the 11 targets set [51]. Two years later, Jones et al. only found partial actions for four targets out of 12. Lindberg et al. (high quality) investigated whether sodium reduction was a priority for processed food manufacturers (*n* = 33) up to 8 years after the FHD launch, by examining their policies and priorities [53]. Half did not provide evidence of sodium reduction actions, and the scope and effectiveness of actions from the other half were reported to be unclear.

The document analysis by Knai et al. on TFA highlighted low levels of participation for the most promising objective: Only 11 of the 90 organizations who had signed up to the TFA reduction pledge committed to fully removing TFAs from their products [43].

As for monitoring, four evaluations underlined issues, including having several to all progress reports being missing or difficult to access [36, 43, 51, 52], delays in progress reports [36, 51], lack of delivery and monitoring plans [36, 51, 52], absence of information about planned monitoring activities by public and voluntary sectors partners [36, 51], lack of systematic baseline data collection [51], and lack of quantitative reporting [51].

### 3.7 Exploration of Heterogeneity and Competing Interests

The findings of studies of human and food environment outcomes may vary by partnership as the effect directions for the FHD are mainly mixed, whereas some of other partnerships are positive, although based on single studies. The results do not appear to vary by study quality, study design, or evaluation period. Three of the five studies that were allocated an effect direction but were not controlled for partnership participation showed a positive effect, highlighting the need for counterfactuals. The number of partnerships without public actors was too small to be compared with PPPs. The five document analyses were rather homogeneous as they were all critical of the same two PPPs.

Three of the five studies reporting positive effects had potential competing interests, including funding by the food industry [37], prior funding and gifts from the food industry [44], and funding and authors from the organization leading the PPP [40]. The study by Beckelman et al., which did not have an effect direction but praised the PPP, was also funded and involved authors from the PPP leading organization (Hawai'i DOH) [39]. Among the four studies with mixed evidence, one involved an author who had received prior funding and gifts from the food industry (the same as above) [45], and another was conducted by researchers affiliated with the PPP leading organization (AWASH) [50]. Two of the document analyses did not include a statement on competing interests [52, 53], and one also did not disclose funding sources [53]. Details are provided in Table S7.

## 4 Discussion

This systematic review of over 10 years of research assessed 17 studies on seven partnerships with commercial actors (particularly the food industry) to improve diets via food reformulation and other changes to the food environment. Evidence was mixed with respect to their impact on purchases or sales of calories and school meals (three studies of low to moderate quality), for reducing product sodium content (six studies of mainly low quality), and for meeting sodium targets (five studies of mainly low quality). Furthermore, there were some positive effects from one uncontrolled study each regarding TFA intake (low quality) and making healthier options more available and visible in school cafeterias (low quality). However, TFA content in food is likely to have started to decline before the partnership (highlighted by a document analysis) [43], and the second study relied on self‐reported data [40]. The five document analyses highlighted limitations in the design, implementation, and monitoring of two PPPs, including limited and vague scopes, a lack of additionality to ongoing actions, and a lack of monitoring and reporting by participants. The number of private organizations committing to the seven partnerships was also highly variable. No study assessed health outcomes or advertising and marketing control initiatives.

Although stemming from only four countries, the results for human and food environment outcomes are similar to those of earlier studies in other countries including Canada, Chile, Germany, the Republic of Korea, Romania, Spain, and the EU [54, 55, 56, 57, 58]. Given the failure of voluntary policy approaches for improving food environments, there are also increasing calls for statutory approaches including taxes on sugar‐sweetened beverages, food marketing to children, food reformulation, and front‐of‐pack labeling [59, 60, 61]. They align with the findings of a systematic review on 25 PPPs to promote health, which reported that PPPs aiming to prevent noncommunicable diseases (NCDs, including public health nutrition interventions) tended to have more negative outcomes than those on infectious disease or other health issues [20]. They also highlighted that independent evaluations of both NCD‐related PPPs and PPPs involving industries with competing commercial interests were rarely positive. For example, the influence of the food industry, which has competing interests, on food and nutrition research has been demonstrated multiple times [62, 63, 64].

Issues in the design, implementation, and monitoring of PPPs are echoed in some of the other included studies. For example, Trevena et al. estimated that the sodium content of pasta sauces would remain too high even if all manufacturers met the FHD target [49]. Ng et al. reported a lack of added value for the HWC since the decline in calories purchased was mainly attributed to external factors and started before the partnership [45]. Beckelman et al. could use only self‐reported purchasing data because the retail partners refused to share their sales data [39].

### 4.1 Implications for Policy and Evaluation

The lack of evidence supporting partnerships with commercial actors to improve food environments might be due to their design resting on a series of unverified assumptions, including that food industry actors are invested in population health, are supportive and compliant in voluntary agreements, and that such partnerships are evidence driven and designed to be equitably managed. Other studies have also challenged these premises [15, 16, 19, 65]. Alternative policy approaches to partnerships with commercial actors who have competing interests should be considered. At least, partnerships should attempt to avoid the limitations identified in our systematic reviews relating to partnership design, implementation, participation, monitoring and evaluation, and competing interests. These limitations are summarized in Table 3 together with examples of questions designed to elicit best practices in partnerships. These questions can guide the planning of both the partnerships and their evaluations.

**TABLE 3:** Reported limitations in partnerships and suggested questions to identify and address these shortcomings.

Category	Limitations	Questions to identify and address the limitations
Design	Limited and vague scope; Focus on less effective actions (or not best practice); No action mentioned; Indicators difficult to measure; Lack of additionality to ongoing actions	Are objectives “SMART”? Are objectives and actions going beyond “business as usual”? Are objectives and actions based on the best evidence of effectiveness? How are the various components of the partnership theorized to work together and in combination to produce effects? Is this clearly spelled out?
Implementation	Lack of commitment; Lack of actions; Absence or inadequate incentives and disincentives; Evaluations not aligned with policy milestones	How many partners have committed to each pledge or objective? Is there a minimum expectation in terms of number or topics on which to commit? How many partners have acted on the committed pledges or objectives? Are the actions relevant? Are there incentives to achieve targets, and are there consequences when missing them? Are the evaluations aligned with key policy milestones?
Participation	Major players missing; “Unequal playing field” for those who join the partnership	Are major actors represented in the partnership? Is there a minimum number of participants expected? Is participation an “equal field” across a given sector? What are the drivers of participation? What are the motivations of actors?
Monitoring	Limited access granted to data; Limited access to monitoring reports; Limited application of sanctions for not complying	Are data reporting and monitoring standards set clearly at the outset? Are there consequences for not complying to the partnership once agreed or for not sharing data? Are incentives for attaining targets, as well as sanctions for not complying, applied? Do they have the intended effect? How have unintended consequences of the partnership been accounted for and reported?
Competing interests	Among the partnership's actors; In the evaluation teams	What means have been taken to avoid or limit the influence of competing interests in the partnership's design, implementation, and monitoring? Are there clear conflicts of interest and power imbalances? Do some study coauthors have potential competing interests (e.g., funding, gifts, nonmonetary advantages, affiliations)? If so, are the results aligned with those of studies that do not have such interests?

Firstly, the objectives and targets of partnerships can be limited, be vague, and focus on approaches known to be less effective and not add value to ongoing actions. Thus, measuring whether targets were met or not can be insufficient as a way of assessing a partnership's effectiveness. Partnerships designed by or with the food industry appear particularly problematic: They can exclude the aspects of companies that negatively impact health the most if they are profitable. The aim of the HWC (selling one trillion fewer calories in the United States by 2012 compared to 2007 and 1.5 trillion fewer by 2015) stands out as it focused on calorie sales rather than calorie content and does not consider product categories. This is challenging to measure and does not indicate specific actions, and an increase in calorie sales is not necessarily problematic: In the study by Ng et al., the sales of calories from fresh and frozen fruit increased by 54% [44]. The choice of 2007 as baseline year is also interesting as it is 2 years before the PPP launch and was followed by an economic recession. It has been argued that the corporations leading the policy were aware that their calorie sales were already declining steadily and that they tried to reduce pressure from advocacy groups and the government by promoting a health halo around this decline using impressive numbers (trillions of calories) [66]. Public and voluntary sector actors need to ensure that the scope, objectives, and targets of partnerships are “SMART” (Specific, Measurable, Achievable, Relevant, and Timebound) and meaningful for population health. Researchers need to, at least, question the content and context of the policy or policies and, at best, appraise them formally and independently.

A second limitation is that commercial partners can commit to few pledges or actions within a partnership and undertake limited activities to achieve them. We also identified mismatches between cutoff dates used in some evaluations and the policy timeline, e.g., using 2008 to differentiate the pre–post HWC periods instead of 2009 or 2010 [45] and evaluating ready‐meal targets before their introduction [50]. From a policy perspective, this suggests the need for greater leadership, monitoring, incentives to achieve targets, and consequences when they are missed. From an evaluation perspective, it highlights the value of documentary analyses for shedding light on policy processes and informing both the design and interpretation of observational studies, like process evaluations can do within trials for assessing complex interventions [67].

Thirdly, given the voluntary nature of partnerships, the number of organizations involved can vary substantially, especially when they can opt in to specific objectives. A low level of participation of major organizations can undermine the efficacy of a partnership [65]. For example, while a higher sodium content in nonparticipating products is depicted as a success, it can also be a failure to attract the right players to the table if the products are popular. Moreover, the absence of key actors can create an “unequal playing field” for those who join in [19]. Some food industry actors said they preferred regulation for that reason, as it established a level playing field [19]. A minimum level of organizational participation should therefore be required in such policies and should be recorded and considered in the evaluations. Assessing the drivers of participation can also help with both designing and evaluating partnerships.

Other limitations are that many targets were not met after several years and that monitoring data were missing or difficult to access for a few PPPs. This highlights the importance of an accountability framework to guide the design of food environment PPPs, such as the one by Kraak et al. and the importance of assessing the implementation of PPPs, their performance, and the application of incentives and disincentives [68]. Data reporting and monitoring standards should be set clearly at the outset, and data on unintended consequences should be reported.

Lastly, our systematic review underlines the importance of identifying and limiting the influence of competing interests in the policy process (including design, implementation, and monitoring) and evaluations. Partnerships have been described as a “bargaining game” in which the policy adopted reflects the preferences of those with most power [16]. When partnerships inevitably involve actors with competing interests, careful attention should be given to control their influence. Although more easily said than done, this can be facilitated by implementing a steering committee without potential competing interests, by developing partnerships based on evidence, and by using a transparent, deliberative, and participatory engagement process involving all stakeholders [68]. Potential competing interests in evaluations should be avoided. In two included studies, some authors were affiliated with the partnership's leading organization. While this “insider” position can provide study authors with a more accurate understanding of the intervention and a better access to internal data, the study by Beckelman et al. showed that this is not always the case as the two store chains refused to share their sales data [39]. The results of studies with potential competing interests need to be considered in the light of independent studies.

Addressing these limitations will require stronger government (or nonprofit organization) leadership with adequate funding and independent resources. Our systematic review also highlights the need to integrate more systematically controls or counterfactuals for partnership participation, to collect baseline data, and to analyze bigger samples of products. Where sample size is small due to few products in a category participating (or not) to a policy, researchers could consider merging them with another category.

### 4.2 Strengths and Limitations of the Systematic Review

To our knowledge, this is the first systematic review of partnerships with commercial actors designed to improve the food environment. While it focused on the state, national, and international levels; included few countries; and was restricted to research conducted between 2010 and 2020, the lessons for policy and evaluation are relevant more broadly. Despite methodological challenges, we consider the inclusion of studies of the food environment and documents as a strength as they helped provide nuance to the results of some studies of human outcomes. The literature search strategy was comprehensive, did not include geographic and language limits, and identified a large number of food environment policies (482 publications were included in our evidence map). Yet, only a few partnerships were found. We do not know if this is due to the presence of few partnerships with commercial actors worldwide to improve food environments at state, national, or international levels; if this is because few of these partnerships have been evaluated; or whether our literature strategy missed these evaluations because the literature strategy did not specify partnership names and focused on academic sources. Our evidence map suggests that the capacity to evaluate food environment policies is limited worldwide as 81% of the 482 included publications assessed the same 12 countries (including the four countries included in this systematic review) [18]. Further studies could identify partnerships a priori and then search for their evaluations in academic and gray literature sources.

Study findings were synthesized by effect direction. This method does not account for the magnitude and size of effects. There were also too few studies of partnerships between the voluntary and private sectors to analyze them separately from PPPs. Regarding study appraisal, instead of using ROBINS‐I (which we piloted on a few studies), we adapted a version of the Newcastle–Ottawa Scale for cross‐sectional studies. ROBINS‐I was designed for follow‐up (cohort) studies of interventions assumed to be planned or managed by researchers to some extent. Our focus on real‐world policies made items about co‐interventions, classification of interventions, and deviations from intended interventions challenging to rate meaningfully. In fact, a poor implementation or deviation from the partnership does not necessarily imply a bias; it can also be an outcome (deliberate or not). Moreover, document analyses are increasingly being considered in evidence synthesis on the commercial determinants of health [69]. Work is needed to develop and validate an appraisal tool that applies to different types of policy evaluations, including studies of the food environment and documents and cross‐sectional health‐related studies [70]. In the absence of such a tool, we adapted an existing tool as critical appraisal is a key step of the systematic review process for considering the robustness of the evidence assessed. We acknowledge that this limits our confidence in judging the strength of the evidence, although this should affect our conclusions to a limited extent as most of the evidence was rated “low quality.” While our tool is not validated, we believe it provides a useful example of how a critical appraisal tool could be applied to these types of policy evaluations. One of our team members is a coauthor of two of our included studies (C.K.). She was involved neither in the development nor in the application of our quality assessment tool.

Lastly, we had planned to use the GRADE framework to assess the level of confidence in the evidence. However, it became apparent that it was developed for different and/or narrower research questions on more homogeneous interventions and outcomes. The appraisal of publication biases and inconsistency was particularly problematic. Furthermore, GRADE does little to differentiate levels of certainty from observational studies: They all are rated as “low” or “very low,” despite being sometimes the best sources that can realistically be obtained. We felt that this led to the production of statements that appeared absolute but were uninformative at best and misleading at worst. This risks sending the message to decision‐makers that there is no good evidence and that they may use non–evidence‐based sources instead. Guidance for policy evaluations and document analyses is needed.

## 5 Conclusion

This systematic review finds that partnerships with commercial actors to improve food environments at the state, national, and international levels have limited effectiveness at achieving this aim, particularly for product sodium reduction and improving the nutritional quality of food purchases. It also highlights several problems with such partnerships, including their limited scope; limited added value; low participation levels; and lack of implementation, monitoring, reporting, and enforcement. These findings have fundamental implications for the design and evaluation of policy interventions to improve the food environment. This work also makes considerable methodological contributions to critical appraisal of policy evaluations. More work needs to be done to continue developing and validating tools for this purpose.

## Ethics Statement

The authors have nothing to report.

## Conflicts of Interests

The authors declare no conflicts of interest.

## Supporting information


**Table S1.** Preferred Reporting Items for Systematic Reviews and Meta‐Analyses (PRISMA) checklist.
**Table S2.** Literature search strategy in MEDLINE (Ovid SP).
**Table S3.** Modifications to the Newcastle–Ottawa Scale for cross‐sectional studies.
**Table S4.** Quality of studies of human outcomes, using a modified version of the Newcastle–Ottawa Scale (*n* = 5).
**Table S5.** Quality of studies of food environment outcomes, using a modified version of the Newcastle–Ottawa Scale (*n* = 6).
**Table S6.** Quality of document analyses, using a modified version of the Newcastle–Ottawa Scale (*n* = 5).
**Table S7.** Study characteristics and results (*n* = 16).
