# Supplementary material for: The Effectiveness of Partnerships With Commercial Actors to Improve Food Environments: A Systematic Review
Source: Obes Rev. 2025 Jun 29;26(10):e13952. doi: 10.1111/obr.13952 (PMC12404887; doi:10.1111/obr.13952)
Supplement: Supplementary file 1 — Table S1. Preferred Reporting Items for Systematic Reviews and Meta‐Analyses (PRISMA) checklist. Table S2. Literature search strategy in MEDLINE (Ovid SP). Table S3. Modifications to the Newcastle–Ottawa Scale for cross‐sectional studies. Table S4. Quality of studies of human outcomes, using a modified version of the Newcastle–Ottawa Scale (n = 5). Table S5. Quality of studies of food environment outcomes, using a modified version of the Newcastle–Ottawa Scale (n = 6). Table S6. Quality of document analyses, using a modified version of the Newcastle–Ottawa Scale (n = 5). Table S7. Study characteristics and results (n = 16). [file OBR-26-e13952-s001.pdf]

Supporting Information

The effectiveness of partnerships with commercial actors to improve food environments: A systematic review

Laurence Blanchard[1], Gemma Bridge[2,3], Julia Bidonde[4], Matt Egan[1], Harry Rutter[5], Mark Petticrew[1,6], Patricia J Lucas[7,8], Monique Potvin Kent[9], Claire Bennet[10], Stephanie Ray[1], Cherry Law[1,11], Cécile Knai[1,6]*

[1] Faculty of Public Health Policy, London School of Hygiene & Tropical Medicine, UK

[2] School of Earth and Environment, University of Leeds, UK

[3] York Business School, York St John University, UK

[4] School of Rehabilitation Science, College of Medicine, University of Saskatchewan, Canada

[5] Department of Social & Policy Sciences, University of Bath, UK

[6] SPECTRUM Consortium, UK

[7] School for Policy Studies, University of Bristol, UK

[8] Colectiv Tech, Bristol, UK

[9] School of Epidemiology and Public Health, University of Ottawa, Canada

[10] Public Health and Wellbeing team, Greenwich Borough, London, UK

[11] Department of Agri-Food Economics and Marketing, University of Reading, UK

*** Corresponding author:** Laurence.blanchard1@lshtm.ac.uk; London School of Hygiene & Tropical Medicine, 15-17 Tavistock Place, London WC1H 9SH, UK.

1. Contents

TABLE S1. PREFERRED REPORTING ITEMS FOR SYSTEMATIC REVIEWS AND META-ANALYSES (PRISMA) CHECKLIST 3

TABLE S2. LITERATURE SEARCH STRATEGY IN MEDLINE (OVID SP) 7

TABLE S3. MODIFICATIONS TO THE NEWCASTLE-OTTAWA SCALE FOR CROSS-SECTIONAL STUDIES 11

TABLE S4. QUALITY OF STUDIES OF HUMAN OUTCOMES, USING A MODIFIED VERSION OF THE NEWCASTLE-OTTAWA SCALE (N=5) 14

TABLE S5. QUALITY OF STUDIES OF FOOD ENVIRONMENT OUTCOMES, USING A MODIFIED VERSION OF THE NEWCASTLE-OTTAWA SCALE (N=6) 15

TABLE S6. QUALITY OF DOCUMENT ANALYSES, USING A MODIFIED VERSION OF THE NEWCASTLE-OTTAWA SCALE (N=5) 15

TABLE S7. STUDY CHARACTERISTICS AND RESULTS (N=16) 16

REFERENCES 24

Table S1. Preferred Reporting Items for Systematic reviews and Meta-Analyses (PRISMA) checklist

This table shows where each item listed in the PRISMA 2020 checklist is reported in the manuscript

Section and Topic	Item #	Checklist item	Location where item is reported
**TITLE**			
Title	1	Identify the report as a systematic review.	Title
**ABSTRACT**			
Abstract	2	See the PRISMA 2020 for Abstracts checklist.	Abstract
**INTRODUCTION**			
Rationale	3	Describe the rationale for the review in the context of existing knowledge.	Introduction
Objectives	4	Provide an explicit statement of the objective(s) or question(s) the review addresses.	End of intro
**METHODS**			
Eligibility criteria	5	Specify the inclusion and exclusion criteria for the review and how studies were grouped for the syntheses.	Eligibility criteria, literature search strategy + Table S2
Information sources	6	Specify all databases, registers, websites, organisations, reference lists and other sources searched or consulted to identify studies. Specify the date when each source was last searched or consulted.	
Search strategy	7	Present the full search strategies for all databases, registers and websites, including any filters and limits used.	literature search strategy + Table S2
Selection process	8	Specify the methods used to decide whether a study met the inclusion criteria of the review, including how many reviewers screened each record and each report retrieved, whether they worked independently, and if applicable, details of automation tools used in the process.	Eligibility criteria
Data collection process	9	Specify the methods used to collect data from reports, including how many reviewers collected data from each report, whether they worked independently, any processes for obtaining or confirming data from study investigators, and if applicable, details of automation tools used in the process.	Data extraction
Data items	10a	List and define all outcomes for which data were sought. Specify whether all results that were compatible with each outcome domain in each study were sought (e.g. for all measures, time points, analyses), and if not, the methods used to decide which results to collect.	Eligibility criteria + Data extraction
	10b	List and define all other variables for which data were sought (e.g. participant and intervention characteristics, funding sources). Describe any assumptions made about any missing or unclear information.	Eligibility criteria + Data extraction

Section and Topic	Item #	Checklist item	Location where item is reported
Study risk of bias assessment	11	Specify the methods used to assess risk of bias in the included studies, including details of the tool(s) used, how many reviewers assessed each study and whether they worked independently, and if applicable, details of automation tools used in the process.	Study quality appraisal
Effect measures	12	Specify for each outcome the effect measure(s) (e.g. risk ratio, mean difference) used in the synthesis or presentation of results.	Eligibility criteria + Data extraction
Synthesis methods	13a	Describe the processes used to decide which studies were eligible for each synthesis (e.g. tabulating the study intervention characteristics and comparing against the planned groups for each synthesis (item #5)).	Data synthesis
	13b	Describe any methods required to prepare the data for presentation or synthesis, such as handling of missing summary statistics, or data conversions.	Mentioned for Dunford's et al study in the results section (for calculating confidence intervals)
	13c	Describe any methods used to tabulate or visually display results of individual studies and syntheses.	Data synthesis
	13d	Describe any methods used to synthesize results and provide a rationale for the choice(s). If meta-analysis was performed, describe the model(s), method(s) to identify the presence and extent of statistical heterogeneity, and software package(s) used.	
	13e	Describe any methods used to explore possible causes of heterogeneity among study results (e.g. subgroup analysis, meta-regression).	
	13f	Describe any sensitivity analyses conducted to assess robustness of the synthesized results.	N/A
Reporting bias assessment	14	Describe any methods used to assess risk of bias due to missing results in a synthesis (arising from reporting biases).	Study quality appraisal
Certainty assessment	15	Describe any methods used to assess certainty (or confidence) in the body of evidence for an outcome.	Not considered but discussed in the discussion
**RESULTS**			
Study selection	16a	Describe the results of the search and selection process, from the number of records identified in the search to the number of studies included in the review, ideally using a flow diagram.	Beginning of results section
	16b	Cite studies that might appear to meet the inclusion criteria, but which were excluded, and explain why they were excluded.	
Study characteristics	17	Cite each included study and present its characteristics.	Characteristics of partnerships & Characteristics

Section and Topic	Item #	Checklist item	Location where item is reported
			of included studies + Tables 1-2+ Table S7
Risk of bias in studies	18	Present assessments of risk of bias for each included study.	Quality appraisal + Tables S4-5-6
Results of individual studies	19	For all outcomes, present, for each study: (a) summary statistics for each group (where appropriate) and (b) an effect estimate and its precision (e.g. confidence/credible interval), ideally using structured tables or plots.	Table 2 and text in the results sections on the three types of outcomes
Results of syntheses	20a	For each synthesis, briefly summarise the characteristics and risk of bias among contributing studies.	Results sections on the three types of outcomes + Table 2
	20b	Present results of all statistical syntheses conducted. If meta-analysis was done, present for each the summary estimate and its precision (e.g. confidence/credible interval) and measures of statistical heterogeneity. If comparing groups, describe the direction of the effect.	N/A
	20c	Present results of all investigations of possible causes of heterogeneity among study results.	Exploration of heterogeneity
	20d	Present results of all sensitivity analyses conducted to assess the robustness of the synthesized results.	N/A
Reporting biases	21	Present assessments of risk of bias due to missing results (arising from reporting biases) for each synthesis assessed.	Study quality appraisal
Certainty of evidence	22	Present assessments of certainty (or confidence) in the body of evidence for each outcome assessed.	N/A
**DISCUSSION**			
Discussion	23a	Provide a general interpretation of the results in the context of other evidence.	Discussion, after the summary of results
	23b	Discuss any limitations of the evidence included in the review.	Strengths and limitations

Section and Topic	Item #	Checklist item	Location where item is reported
	23c	Discuss any limitations of the review processes used.	Strengths and limitations
	23d	Discuss implications of the results for practice, policy, and future research.	Implications for policy and evaluation
**OTHER INFORMATION**			
Registration and protocol	24a	Provide registration information for the review, including register name and registration number, or state that the review was not registered.	Abstract + start of Methods
	24b	Indicate where the review protocol can be accessed, or state that a protocol was not prepared.	Start of Methods
	24c	Describe and explain any amendments to information provided at registration or in the protocol.	Start of Methods + Discussion (strengths and limitations)
Support	25	Describe sources of financial or non-financial support for the review, and the role of the funders or sponsors in the review.	Title page
Competing interests	26	Declare any competing interests of review authors.	Title page
Availability of data, code and other materials	27	Report which of the following are publicly available and where they can be found: template data collection forms; data extracted from included studies; data used for all analyses; analytic code; any other materials used in the review.	Not reported

From: Page MJ, McKenzie JE, Bossuyt PM, Boutron I, Hoffmann TC, Mulrow CD, et al. The PRISMA 2020 statement: an updated guideline for reporting systematic reviews. BMJ 2021;372:n71. doi: 10.1136/bmj.n71.
http://www.prisma-statement.org/

Table S2. Literature search strategy in MEDLINE (Ovid SP)

The search strategy contains eight key search lines (or eight main concepts) which are highlighted in pale grey and combined in the final search line. For further information, please consult the original report:

L. Blanchard, S. Ray, C. Law, M.J. Vega-Sala, J. Bidonde, G. Bridge, et al., The effectiveness, cost-effectiveness and policy processes of regulatory, voluntary and partnership policies to improve food environments: an evidence synthesis, Public Health Res. (Southampt). 12 (8) (2024) 1–173, https://doi.org/10.3310/JYWP4049.

1 exp Diet/

2 exp Food/

3 beverages/ or exp artificially sweetened beverages/ or exp carbonated beverages/ or exp coffee/ or exp drinking water/ or exp energy drinks/ or exp "fruit and vegetable juices"/ or exp milk/ or exp milk substitutes/ or exp sugar-sweetened beverages/

4 exp Fruit/

5 exp Vegetables/

6 exp Sodium, Dietary/

7 exp Sugars/

8 exp Fats/

9 exp Dietary Fiber/

10 exp Portion Size/ or exp Serving Size/

11 exp Infant Food/ or exp Infant Formula/

12 (Diet or Nutrition or Food or foods or Snack or snacks or Drink or drinks or Beverage* or Soda or sodas or Fruit or fruits or Vegetable* or Salt or Sodium or Sugar* or Fat or fats or fatty acids or TFAs or Fibre or fibres or fiber or fibers or "Portion size*" or "Serving size*" or Menu or menus or Infant formula or infant formulas or baby formula or baby formulas or baby milk or infant milk or artificial milk or breastmilk substitute* or breast milk substitute*).ti,ab.

13 1 or 2 or 3 or 4 or 5 or 6 or 7 or 8 or 9 or 10 or 11 or 12 ***[Food free + MeSH terms]***

14 exp Legislation, Food/ ***[semi-final line 1; for food policies that are clearly regulatory]***

15 (Law or laws or Legislat* or Regulat* or Decree or "Executive order" or Tax or taxes or taxation or taxed or taxing or Levy or levies or levied or "Excise duty" or "fiscal policy" or "fiscal policies" or "fiscal measure" or "fiscal measures").ti,ab. ***[terms related to policies that are clearly regulatory]***

16 ((Law or laws or Legislat* or Regulat* or Decree or “Executive order” or Tax or taxes or taxation or taxed or taxing or Levy or levies or levied or “Excise duty” or “fiscal policy” or “fiscal policies” or “fiscal measure” or “fiscal measures”) adj5 (Diet or Nutrition or Food or foods or Snack or snacks or Drink or drinks or Beverage* or Soda or sodas or Fruit or fruits or Vegetable* or Salt or Sodium or Sugar* or Fat or fats or fatty acids or TFAs or Fibre or fibres or fiber or fibers or “Portion size*” or “Serving size*” or Menu or menus or Infant formula or infant formulas or baby formula or baby formulas or baby milk or infant milk or artificial milk or breastmilk substitute* or breast milk substitute*)).ti,ab. ***[semi-final line 2; 15 adj5 12, for policies that are regulatory + food free terms]***

17 exp Fiscal Policy/ or exp Taxes/

18 exp Government Regulation/

19 17 or 18 ***[MeSH terms associated with policies that are regulatory or about governance]***

20 19 and 13 ***[semi-final line 3; MeSH policies that are regulatory + food]***

21 (“Public-private partnership*” or “Responsibility Deal”).mp. ***[terms clearly related to PPPs]***

22 exp Public-Private Sector Partnerships/

23 21 or 22 ***[free key words + MeSH clearly about PPP]***

24 23 and 13 ***[semi-final line 4; clearly PPP + food]***

25 (Regulatory or Compulsory or Obligat* or obliged or Voluntary or Option* or Non-compulsory or Non-regulatory or Non-obligatory or Public-Private).mp. ***[terms related to governance]***

26 exp Regulatory Reporting/ or exp Regulatory Programs/

27 exp Voluntary Programs/

28 25 or 26 or 27 ***[free and MeSH terms related to governance]***

29 exp Nutrition Policy/

30 exp Food Labeling/

31 exp Food Assistance/

32 29 or 30 or 31 ***[policies that are clearly about food]***

33 28 and 32 ***[semi-final line 5; governance + food-related policies]***

34 (Government* or Governance or Minist* or Senate or ((National or federal or state or provincial) adj (department or agency or institute))).ti,ab. ***[free words related to the national or state public sector]***

35 government/ or exp federal government/ or exp government agencies/ or exp state government/

36 (Industry or industries or Private or Business* or Public-private or Company or companies or Corporat* or Multinational* or Vendor* or Retail* or Shop or shops or Store or stores or supermarket* or Restaura* or Broadcaster*).ti,ab. ***[free terms related to relevant private sectors]***

37 exp Food-Processing Industry/ or exp Food Industry/

38 exp Restaurants/

39 exp Food Services/

40 34 or 35 or 36 or 37 or 38 or 39 ***[free and MeSH terms about the public and private sectors]***

41 (Policy or policies or Plan or Strategy or strategies or Standard or standards or Scheme* or Program* or Guide or guides or guidance or guidelines or Code or codes or Measure or Measures or Rulebook or Target or targets or Limit or limits or limitation or Reformulat* or Remov* or Restrict* or Prohibit* or Ban or bans or banned or Label* or Population intervention* or population-level intervention* or population-based intervention*).ti,ab. ***[free terms frequently used to name diet-related policies]***

42 exp Policy Making/

43 41 or 42 ***[free and MeSH terms about policy]***

44 13 and 28 and 40 and 43 ***[semi-final line 6; food + governance + public/private actors + policy]***

45 (Agreement* or Alliance* or Coalition* or Collaboration or Cooperation or "Joint deliver*" or Partnership* or Pledge* or Self-regulat*).ti,ab. ***[free terms related to partnership]***

46 ((Agreement* or Alliance* or Coalition* or Collaboration or Cooperation or "Joint deliver*" or Partnership* or Pledge* or Self-regulat*) adj5 (Industry or industries or Private or Business* or Public-private or Company or companies or Corporat* or Multinational* or Vendor* or Retail* or Shop or shops or Store or stores or supermarket* or Restaura* or Broadcaster*)).ti,ab. ***[45 adj5 36, to identify partnerships with private actors free terms]***

47 46 and 13 ***[semi-final line 7; partnerships with private actors + food]***

48 ("policy option" or "policy options").mp.

49 48 and 13 ***[semi-final line 8; policy options + food]***

Keywords excluded

50 14 or 16 or 20 or 24 or 33 or 44 or 47 or 49 ***[combination of the 8 strategies]***

51 exp Pharmacology/

52 exp Food Safety/

53 exp Hygiene/

54 exp Food Hypersensitivity/

55 exp Genetics/

56 exp Toxicology/

57 exp cell physiological phenomena/ or exp genetic phenomena/ or exp microbiological phenomena/

58 exp heterocyclic compounds/ or exp polycyclic compounds/ or exp macromolecular substances/ or exp "hormones, hormone substitutes, and hormone antagonists"/ or exp "enzymes and coenzymes"/ or exp "nucleic acids, nucleotides, and nucleosides"/ or exp complex mixtures/ or exp biological factors/ or exp "biomedical and dental materials"/

59 (Cell* or mitochondr* or abell* or mononucl* or nucle* or reductase or abellin* or oxydat* or oxidase or homeostas* or overexpress* or phenotype* or embryo* or abellinge* or PCR or RNA or gene or genes or genetic* or ((calcium or salt or sodium) adj2 ion)).mp.

60 exp animals/ not humans/

61 exp Animal Experimentation/

62 exp Hydrocarbons/

63 exp Forensic Genetics/

64 exp pharmacologic actions/

65 exp plant extracts/ or exp prescription drugs/

66 exp Drug Therapy/

67 exp Biopharmaceutics/

68 51 or 52 or 53 or 54 or 55 or 56 or 57 or 58 or 59 or 60 or 61 or 62 or 63 or 64 or 65 or 66 or 67

69 50 not 68

70 limit 69 to yr="2000 -Current"

71 exp address/ or exp bibliography/ or exp biography/ or exp collected work/ or exp collection/ or exp comment/ or exp congress/ or exp dataset/ or exp dictionary/ or exp directory/ or exp editorial/ or exp guideline/ or exp lecture/ or exp letter/ or exp news/ or exp newspaper article/ or exp overall/ or exp periodical index/ or exp video-audio media/ or exp webcast/

Final search line

72 70 not 71

Table S3. Modifications to the Newcastle-Ottawa Scale for cross-sectional studies

All the modifications that we have made to the tool are explained in the table below and shown in red, except for the quality ratings which are shown in green, amber, dark red and grey.

Categories & items	Original tool for cross-sectional studies*	Adapted tool for this project (modifications in *red*)
Selection		
Representativeness of the sample (search strategy for document analyses)	a. Truly representative of the average in the target population. * (all subjects or random sampling)<br>b. Somewhat representative of the average in the target group. * (non-random sampling)<br>c. Selected group of users/convenience sample.<br>d. No description of the derivation of the included subjects.	a. Truly representative of the average in the target population (all subjects or random sampling) (high quality)<br>b. b. Somewhat representative of the most common units, e.g., the most popular TV channels, in the target group. (non-random sampling;) (moderate quality)<br>c. Selected group of users/convenience sample. (low quality)<br>d. No description of the derivation of the included subjects. (no info/unclear)<br><br>Document analyses: we considered instead the literature search (how comprehensive and clear it was)
Sample size (variety of information sources for document analyses)	a. Justified and satisfactory (including sample size calculation). *<br>b. Not justified.<br>c. No information provided	a. Justified in a satisfactory manner ~~(including sample size calculation).~~ (high quality)<br>b. No appropriate justification. (moderate or low quality; case by case)<br>c. No information provided (no info/unclear)<br><br>Document analyses: we considered instead the variety of information sources used, taking into account the study aim.
Non-respondents	a. Proportion of target sample recruited attains pre-specified target or basic summary of non-respondent characteristics in sampling frame recorded. *<br>b. Unsatisfactory recruitment rate, no summary data on non-respondents.<br>c. No information provided	a. Proportion of target sample recruited attains pre-specified target or basic summary of non-respondent characteristics in sampling frame recorded. (high quality)<br>b. Unsatisfactory recruitment rate, no summary data on non-respondents. (low quality)<br>c. No information provided (no info/unclear)<br><br>Studies of food environment outcomes and document analyses: not applicable<br>d.
Missing data		New item<br>a. Methods to deal with missing data are explained and appropriate (high quality)<br>b. Methods to deal with missing data unclear or inappropriate (moderate or low quality; case by case)

Ascertainment of the exposure (risk factor)	a. Vaccine records/vaccine registry/clinic registers/hospital records only. **<br>b. Parental or personal recall and vaccine/hospital records. *<br>c. Parental/personal recall only.	a. No obvious reason to believe that they did not use an up-to-date list of participants or that participant status changed during the evaluation period and was not accounted for, using info in the evaluations and policy documents (high quality).<br>b. Reasons to believe that the list of participants was not up to date or that membership is likely to have changed during the evaluation period and was not considered in the evaluation, using info in the paper & policy in general (low quality).<br>c. No information provided (no info/unclear)<br><br>Studies of food environment outcomes: Studies that aggregate data on participants and non-participants together were given a low-quality rating and the remaining studies were judged as moderate.<br><br>Document analyses: not applicable.
Comparability		
Comparability of subjects in different outcome groups on the basis of design or analysis. Confounding factors controlled.	a. Data/ results adjusted for relevant predictors/risk factors/confounders e.g. age, sex, time since vaccination, etc. **<br>b. Data/results not adjusted for all relevant confounders/risk factors/information not provided.	a. Data/ results adjusted for relevant predictors/risk factors/confounders (high quality)<br>b. Studies of food environment outcomes controlled for PPP participation (moderate)<br>c. Data/results not adjusted for all relevant confounders/risk factors/information not provided, or study not controlled for PPP participation (including studies of PPP participants only, studies that aggregate data from both participant and participants, and studies controlled for other factors than PPP participation). (low quality)<br><br>Document analyses: not applicable.
Outcome		
Assessment of outcome	a. Independent blind assessment using objective validated laboratory methods. **<br>b. Unblinded assessment using objective validated laboratory methods. **<br>c. Used non-standard or non-validated laboratory methods with gold standard. *<br>d. No description/non-standard laboratory methods used.	a. Blinded AND Double/Single independent assessment using methods appropriate for study aim (high quality)<br>b. Unblinded double/single data (database) (high quality)<br>c. Unblinded double data (collected on the field) (moderate quality)<br>d. Unblinded AND Single using methods appropriate for study aim or unclear (low quality).

Statistical test	a. Statistical test used to analyse the data clearly described, appropriate and measures of association presented including confidence intervals and probability level (p value). *<br>b. Statistical test not appropriate, not described or incomplete.	a. Statistical test used to analyse the data clearly described, appropriate and measures of association presented including confidence intervals and probability level (p value).* (high quality)<br>b. Statistical test not appropriate~~, not described or incomplete~~. (low quality)<br>c. Statistical test ~~not appropriate,~~ not described or incomplete. (no info/unclear)<br><br>Document analyses: consider the analytical methods in general, including descriptive approaches.
Overall rating		
	* = 1 point<br>** = 2 points<br>Very Good Studies: 9-10 points<br>Good Studies: 7-8 points<br>Satisfactory Studies: 5-6 points<br>Unsatisfactory Studies: 0 to 4 points	New classification: 2 key items: 4. ascertainment of exposure & 7. statistical tests<br><br>High quality:<br>• items 4 or 7 rated high, few other items rated moderate, no item rated low<br><br>Moderate quality:<br>• items 4 or 7 rated moderate, and none rated low<br>• judgment based on the other item ratings<br><br>Low quality:<br>• when 1+ item is rated low<br><br>Unclear quality:<br>• items 4 or 7 rated unclear, and none rated low<br>• 2+ items rated unclear, and none rated low

*Herzog R, Álvarez-Pasquin MJ, Díaz C, Del Barrio JL, Estrada JM, Gil Á. Are healthcare workers' intentions to vaccinate related to their knowledge, beliefs and attitudes? a systematic review. BMC Public Health. 2013;13(1)

Table S4. Quality of studies of human outcomes, using a modified version of the Newcastle-Ottawa Scale (n=5)

Author (date)	Representa-tiveness of sample size	Sample size justification	Non-response	Missing data	Ascertain-ment of exposure	Confounders	Assessment of outcome	Statistical test	Overall study quality
Beckelman et al (2020)[39]	Low (-)	Low (-)	Low (-)	Unclear (?)	High (++) (all exposed)	Low (-) (P only)	Low(-)	High (++)	**Low (-)**
Hutchinson et al (2018)[51]	High (++)	Low (-)	Unclear (?)	Unclear (?)	N/A (P & NP combined)	Low (-) (P & NP combined)	High (++)	High (++)	**Low (-)**
Ng & Popkin (2014)[55]	High (++)	High (++)	Unclear (?)	Moderate (+)	High (++)	Moderate (+)	High (++)	High (++)	**Moderate (+)**
Ng et al (2014)[56]	High (++)	High (++)	Unclear (?)	High (++)	High (++)	Moderate (+)	High (++)	High (++)	**Moderate (+)**
Rajbhandari-Thapa et al (2017)[40] *(school meal purchases)*	High (++)	High (++)	Low (-)	Low (-)	High (++)	Low (-)	High (++)	High (++)	**Low (-)**

N/A: Not applicable; NP: Non-participants in the PPP; P: Participants in the PPP. The reference numbers are the same as those employed in the main manuscript.

Table S5. Quality of studies of food environment outcomes, using a modified version of the Newcastle-Ottawa Scale (n=6)

Author (date)	Representative-ness of sample size	Sample size	Missing data	Ascertainment of exposure	Confounders	Assessment of outcome	Statistical test	Overall study quality
Christoforou et al (2013)[45]	Moderate (+)	Unclear (?)	Unclear (?)	N/A (P & NP combined)	Low (-) (P & NP combined)	Low (-)	High (++)	**Low (-)**
Dunford et al (2011)[37]	Low (-)	Unclear (?)	Unclear (?)	N/A (P & NP combined)	Low (-)(P & NP combined)	Low (-)	High (++)	**Low (-)**
Levi et al (2018)[34]	Moderate (+)	High	Unclear (?)	Unclear (?)	Moderate (+)	High	Low (-)	**Low (-)**
Sparks et al (2018)[46]	Moderate (+)	Unclear (?)	Unclear (?)	Unclear?	Moderate (+)	Low (-)	High (++)	**Low (-)**
Trevena et al (2014)[47] *pasta*	Moderate (+)	Unclear (?)	High (++)	N/A (P & NP combined)	Low (-) (P & NP combined)	Low (-)	High (++)	**Low (-)**
Trevena et al (2014)[41] *bread, cereal, meat*	Moderate (+)	Unclear (?)	High (++)	High (++)	Moderate (+)	High (++)	High (++)	**Moderate (+)**
Rajbhandari-Thapa et al[40] *(changes to cafeterias)*	Low (-)	Low (-)	Low (-)	High (++) (all exposed)	Low (-) (P only)	Low (-)	High (++)	**Low (-)**
Robinson et al (2019)[42]	Moderate (+)	Unclear (?)	Unclear (?)	High (++)	Moderate (+)	Moderate (+)	Unclear (?)	**Unclear (?)**

N/A: Not applicable; NP: Non-participants in the PPP; P: Participants in the PPP. The reference numbers are the same as those employed in the main manuscript.

Table S6. Quality of document analyses, using a modified version of the Newcastle-Ottawa Scale (n=5)

Author (date)	Search strategy	Variety of information sources*	Missing data	Assessment of outcome	Analytical methods	Overall study quality
Elliot et al (2014)[58]	Low (-)	Moderate (+)	Unclear (?)	Low (-)	Moderate (+)	**Low (-)**
Jones et al (2016)[36]	High (++)	High (++)	High (++)	Low (-)	Moderate (+)	**Low (-)**
Lindberg et al (2017)[49]	High (++)	High (++)	Unclear (?)	High (++)	High (++)	**High (++)**
Knai et al (2015)[43]	High (++)	High (++)	High (++)	High (++)	High (++)	**High (++)**
Knai et al (2017)[52]	High (++)	High (++)	Unclear (?)	High (++)	High (++)	**High (++)**

* taking into account the research question. The reference numbers are the same as those employed in the main manuscript.

Table S7. Study characteristics and results (n=16)

The studies are organised by alphabetical order within each partnership category. The reference numbers are the same as those employed in the main manuscript.

**Lead author (year)** ***PPP; study quality***	**Study aim**	**Study design (Data collection dates)**	**Sample and data sources**	**Outcomes assessed**	**Results**	**Reported affiliations, funding, and competing interests (CI)**
**AUSTRALIA AND NEW-ZEALAND (n=8)**						
Christoforou et al (2013)[45]<br><br>*AWASH Drop the Salt!*<br><br>*Low (-)*	To define changes in sodium (Na) levels within the ready meal market in AUS between 2008 and 2011.	Repeat cross-sectional (post-post)<br><br>(2008, 2009, 2010, 2011)	Ready meal products: n=107 (2008), 313 ('09), 219 ('10), 265 ('11).<br><br>Labels collected from 2 major and 3 smaller stores (96% of AUS grocery market)	Mean Na content per 100g<br>Compared overall, by ready meal type, and by manufacturer	From 2008-2011, overall Na content of ready meal products was largely unchanged (from 279 to 277 mg)<br>The proportion of AUS ready meals meeting the AWASH target fell slightly over the study period (59% to 57%)<br>Differences between the results achieved by different companies.	Affiliations: University, George Institute for Global Health, AWASH;<br>Funding: AWASH, university, AUS Research Council;<br>CI: NR
Dunford et al (2011)[37]<br><br>*FHD and NZHF*<br><br>*Low (-)*	To define the effectiveness of recent efforts by FHD*, and the NZHF to reduce Na levels in breads in AUS and NZ.<br><br>*The article says AWASH, but they have used the FHD targets	Repeat cross-sectional (pre-post)<br><br>(2007 and 2010)	Packaged sliced bread labels.<br>AUS: n=94 in 2007, n=98 in 2010<br>NZ: n=63 in 2007, n=68 in 2010<br><br>Data from product labels in two major supermarket chains in each country	Mean sodium (Na) content per 100g, compared overall, by bread type, by manufacturer, and between nations.	From 2007-2010, mean Na content of breads in AUS did not change: 434mg/100 g in 2007 vs. 435 mg/100 g in 2010; but a 7% reduction in NZ: 469 mg/100 g and 439 mg/100 g.<br>The proportion of AUS breads meeting the national target increased from 29% in 2007 to 50% in 2010; the proportion of NZ breads meeting the national target increased from 49% in 2007 to 90% in 2010.	Affiliations: University, George Institute for Global Health, AWASH;<br>Funding: AUS NHMRC, AWASH, AUS Food and Grocery Council, New South Wales Health, New South Wales Food Authority;<br>CI: AWASH, UK Food Standards Agency.
Elliot et al (2014)[58]<br><br>*FHD*<br><br>*Low (-)*	To evaluate whether the FHD, is having an impact on reducing premature death and disability caused by unhealthy diet in AUS.	Policy document analysis<br><br>(October 2009 to Sept 2013)	Data on processed foods obtained from a food composition database.<br><br>Data from the FHD website, media releases,	Assessment of the FHD achievements (goals, targets, actions and health outcomes) by adopting the RE-AIM framework (reach,	Data available to evaluate the FHD was limited. Several reports on achievements were missing.<br>Only 11 objectives had been set out of 124 potential actions identified, and none of the 11 were due to have been met by September 2013.<br>8 product categories had set Na targets, . One product category had saturated fat targets, 2	Affiliations: University, George Institute for Global Health, Cancer Council Victoria, WHO, AWASH;<br>Funding: AUS Research Council, AUS NHMRC

Lead author (year) *PPP; study quality*	Study aim	Study design (Data collection dates)	Sample and data sources	Outcomes assessed	Results	Reported affiliations, funding, and competing interests (CI)
			communiqués and e-newsletters	efficacy, implementation and maintenance.<br>Na, saturated fat, added sugar, energy, fibre, whole grains, fruit/vegetable content, portion size.	product categories had portion size targets set, only one had a saturated fat target, and none had targets for added sugar, energy, fibre, whole grains, and fruit and vegetables content. Engagement of relevant companies across product categories ranged from 60 to 100%.	CI: AUS NHMRC, travel reimbursement and honoraria from PepsiCo; AWASH, and other non-industry involvement
Jones et al (2016)[36]<br><br>*FHD*<br><br>*Low (-)*	To evaluate FHD over 6 years and to use the findings to develop recommendations for the success of the new HFP.	Policy document analysis<br><br>(Oct 2013 to Nov 2015)	137 areas of possible FHD action.<br><br>Data from FHD website, media releases, communiqués, e-newsletters, materials released under freedom-of-info, and Parliamentary Hansard.	Assessment of the FHD (goals, targets, actions and health outcomes) by adopting the RE-AIM framework (reach, efficacy, adoption, implementation and maintenance).	Limited data is available to assess achievements. No info about progress towards milestones published since October 2013. Activities were recorded for only 12 of the 137 identified actions.	Affiliations: University, George Institute for Global Health, WHO;<br>Funding: university, AUS Research Council;<br>CI: declared having none
Levi et al (2018)[34]<br><br>*FHD*<br><br>*Unclear (?)*	To examine the food industry's progress and compliance with the FHD Na reduction targets for soup.	Repeat cross-sectional (post-post)<br><br>(between Aug-Dec in 2011, 2012, 2013, 2014)	1153 dry, canned, and chilled soups from the same four grocery retail stores in Sydney, Australia. P products in 2011: 59 (87%) dry soups and 124 (66%) wet soups from 5 companies.<br><br>N companies (NR)<br>Nutrition info from the George Institute	Na content of 'wet and condensed' and of 'dry' soups 'as consumed'.	In 2014, 67% of dry soups and 75% of wet soups met the Na reduction targets.<br>Dry soups: Median sodium values sig decreased from FHD P ($p<0.05$), not for NP. It was lower among P products (no clear values provided). The proportion of P dry soups meeting the target increased from 38% in 2011 to 67% in 2014, but there was not sig. difference with matched NP.<br>Wet soups: Median sodium values decreased from FHD P but this was not stat sign. No improvement among NP. Minor improvement in the proportion of P wet soups meeting the target, from 70% in 2011 to 76% in 2014. When	No apparent CoI.<br>Affiliations: universities<br>Funding: NHMRC;<br>CI: declared having none

**Lead author (year)** *PPP; study quality*	**Study aim**	**Study design (Data collection dates)**	**Sample and data sources**	**Outcomes assessed**	**Results**	**Reported affiliations, funding, and competing interests (CI)**
			Branded Food Composition Database.		comparing 2011 and 2014, there was no difference by status among both NP (matched, p=0.35 and unmatched, p=0.74) and P (matched, P=0.32).<br>A large proportion of products already met the targets in 2011, suggesting that the targets were too low.	
Lindberg et al (2017)[49]<br><br>*FHD*<br><br>*High (++)*	To explore if Australia's largest food manufacturers made positive (nutrition) changes to their product portfolios as disclosed in their public policies, priorities, and communications. To assess if sodium reduction is a priority for them.	Policy document analysis<br><br>(2010 to May 2017)	Grey literature by 33 Australian food manufacturers producing product lines of relevance to salt-reduction: websites, media releases, policies, annual reports and emails.	Evidence of priority and progress for the FHD sodium targets.	All (n=33) manufacturers state nutrition and healthy eating as part of their policies, protocols and priorities.<br>Many (n=16) manufacturers provide no evidence or documentation of reducing sodium in their products.<br>Half of the sample (n=17) describe some sodium reduction activities, and the scale and efficacy of these changes is unclear.	Affiliations: University;<br>Funding: NR except stating that the funder had no role in the study;<br>CI: NR
Sparks et al (2018)[46]<br><br>*FHD*<br><br>*Unclear (?)*	To assess the median Na sodium levels of 2510 processed meat products, including bacon and sausages, available in major Australian supermarkets in 2010, 2013, 2015 and 2017, and assessed changes over time.	Repeat cross-sectional (post-post)<br><br>(2010, 2013, 2015 and 2017)	2510 processed meat products in four major supermarkets in Australia<br>2010: 181 (43%) P;<br>2017: 236 (35%) P.<br><br>Nutrition data from the Australian FoodSwitch database	Na content of processed meat, of which 5 product categories have targets (P): bacon, ham/cured meat products, emulsified luncheon meats, wet savoury pasties and dry savoury pasties	Median Na of P processed meats decreased by 11% (p<0.001) vs. no change in NP (median difference 6%, p=0.450). It was 1010 mg/100 g in 2010 vs. 898 mg/100 g in 2017 for P and 765 mg/100 g in 2010 vs. and 717 mg/100 g in 2017 for all processed meat.	Affiliations: University, Victorian Health Promotion Foundation, Heart Foundation;<br>Funding: NHMRC, National Heart Foundation, WHO, VicHealth, AUS National Health and Medical Research Council;<br>CI: WHO.
Trevena et al (2014)[41]<br><br>*FHD*	To assess Na reduction targets for breads, ready-to-eat breakfast cereals and processed meats. To quantify the magnitude of any changes	Repeat cross-sectional (post-post)	Bread: n=145 (84%) P in 2010; n=177 (66%) P in 2013;	Mean Na content.<br><br>Products included bread products; value added	Mean Na content of bread products and breakfast cereals sig decreased from 454 to 415 mg/100 g and 316 to 237 mg/100 g (p<0.001) respectively. Smaller but still sig reduction in Na in cured meats, bacon, ham (1215 to 1114	Affiliations: University, hospital, George Institute for Global Health, AWASH;

Lead author (year) *PPP; study quality*	Study aim	Study design (Data collection dates)	Sample and data sources	Outcomes assessed	Results	Reported affiliations, funding, and competing interests (CI)
Moderate (+)	in Na content and to explore whether changes differed by manufacturer or product category.	(July and Sept, 2010-2013)	Bacon/ham/cured meat: n=83 (92%) P in 2010; n=98 (88%) P in 2013; Breakfast cereals; n=86 (70%) P in 2010; n=107 (67%) P in 2013.<br><br>Data from product labels in four supermarket chains in Sydney.	products; ready to eat breakfast cereals; and processed meats (bacon/ham/cured meat).	mg/100 g, p=0.001). However, no apparent diff in Na reductions between P and P brands for all three categories.<br>More P bread and breakfast cereals met the targets than non-P. No difference by status for processed meats.<br>Data on emulsified meat were collected but samples were too small (n=9 2010, n=7 2013).	Funding: AUS NHMRC, AUS Research Council.<br>CI: AWASH
Trevena et al (2014)[47]<br><br>*FHD*<br><br>*Low (-)*	To assess change in Na content of AUS pasta sauces between 2008 and 2011.<br>To project the mean Na content of products in 2014, comparing to the 2012 UK Na target for pasta sauce (330mg/100g).	Repeat cross-sectional (post-post)<br><br>(2008 and 2011)	Pasta sauces sold in 5 supermarkets in Sydney, AUS (n=124 sauces in 2008, n=187 in 2011)<br><br>Data from product labels in five leading grocery chains in Sydney	Mean Na content. Manufactures grouped by type according to % volume share in 2009 (supermarket own label; leading manufacturers; other manufacturers).<br>Na content assessed from product labels.	Mean Na content was not sig different between 2008 and 2011 (451 mg/100 g v. 423 mg/100 g; p=0.16).<br>Projected means exceeded 2012 UK target (Scenario 1: 381 mg/100 g; scenario 2: 375 mg/100 g. 22% reduction needed from 2011 levels to meet target.	Affiliations: University, George Institute for Global Health, AWASH;<br>Funding: AWASH, university, AUS NHMRC, AUS National Heart and Stroke Foundation, AUS Research Council;<br>CI: AWASH, UK Food Standards Agency
**UK (n=4)**						
Hutchinson et al (2018)[51]<br><br>*PHRD*<br><br>*Low (-)*	To explore whether voluntary reformulation results in different intakes of trans-fatty acids among socio-economic groups.	Repeat cross-sectional (pre-post)<br><br>(Pre-reformulation: 2000/01 vs. post-reformulatio	UK adults aged 19–64 years. Pre-reformulation: n=1724; Post: n=848<br><br>Data from UK National Diet and Nutrition Surveys. Pre-reformulation: 7-day	TFA intake pre and post voluntary reformulation with comparisons by income, education and disability, and between high and low TFA consumers	Pre-reformulation: 57% of adults exceeded the WHO TFA intake limit, compared with 2.5% post-ref.<br>Pre-reformulation: High TFA intake was associated with lower income, lower education attainment and long-term illness. TFAs were mostly from artificial sources.<br>Post-reformulation: People with higher income were 2.5-3.3 times more likely to be top 10%	Affiliations: University, WHO<br>Funding: WHO;<br>CI: Last author is director and shareholder of myfood24 (online dietary assessment tool).

Lead author (year) *PPP; study quality*	Study aim	Study design (Data collection dates)	Sample and data sources	Outcomes assessed	Results	Reported affiliations, funding, and competing interests (CI)
		n: 2010/11–2011/12)	weighed records; post: 4-day diaries.		consumers. TFAs from ruminant sources were more prominent.	
Knai et al (2015)[43]<br><br>*PHRD*<br><br>*High (++)*	To evaluate the PHRD effectiveness at encouraging signatory organisations to remove artificially produced TFAs from their products.	Policy document analysis<br><br>(2015)	Publicly available data in the progress reports of the 90 signatory organizations to the pledge confirming non-use of TFAs	Compared progress reports against what had been originally set by organizations in their delivery plans.	91% of the signatory organizations (n=82) had already removed TFAs or had removal of TFAs underway before the PHRD started.<br>9% (n=11) signatory organizations signed the pledge to remove artificial TFA from food, of which 5 were not in a position to reformulate food products at the point of manufacture, some reported replacing products.	Affiliations: University;<br>Funding: England NIHR Policy Research Program;<br>CI: Declared having none.
Knai et al (2017)[52]<br><br>*PHRD*<br><br>*High (++)*	To analyse the evidence of the effectiveness of the specific interventions in the PHRD pledges and the likelihood that the pledges have brought about actions among organisations that would not otherwise have taken place.	Policy document analysis (synthesis of PHRD website reports)<br><br>(At implementation: 2011; post: 2015)	253 P in the 6 PHRD food pledges.<br><br>Comparative analysis of published progress reports and publicly available data on PHRD; evidence on effectiveness from an overview of 17 reviews.	Likely effectiveness and added value of pledges.<br>Explored changes over time, and compared to what was originally set out in delivery plans	Progress reports were very inconsistently provided on the PHRD website and mostly unavailable.<br>Some of the PHRD food interventions could be effective, if fully implemented, but there is an emphasis on education and information awareness, which tend to be less effective than structural information.<br>Most interventions reported by organisations seemed either clearly (37%) or possibly (37%) already underway.	Affiliations: University;<br>Funding: England NIHR Policy Research Unit;<br>CI: NR
Robinson et al (2019)[42]<br><br>*PHRD*<br><br>*Unclear (?)*	To examine…<br>2. The proportion of major UK restaurant (fast food and full service) and takeaway chains that provide kcal info at point of choice and compliance with PHRD kcal labelling recommendations;<br>3. The adequateness of kcal labelling practises among eligible chains that also	Cross-sectional (post, once)<br><br>August 2018	Food and beverage items from 104 large chains (16 P; 88 NP).<br>Full-service restaurant chains (n=58)<br>Takeaway or fast-food chains (n=22)<br>Coffee shops (n=20)<br>Supermarket chains (n=4)	Presence of calorie labelling and adherence to policy guidelines.	Of the 104 chains, 18 displayed calorie labelling in stores: 12 (67%) of participants, and 6 (7%) of NPs.<br>None fulfilled all seven recommended labelling criteria.<br>Only one P and 2 NP chains provided calorie labelling for all items sold. 4 P did not display any.<br>43/86 (50%) of the chains that did not provide in store kcal labelling had product kcal info on	Affiliations: University & NR;<br>Funding: UK Medical Research Council;<br>CI: Lead author has worked on research projects funded by American Beverage Association and Unilever;

Lead author (year) *PPP; study quality*	Study aim	Study design (Data collection dates)	Sample and data sources	Outcomes assessed	Results	Reported affiliations, funding, and competing interests (CI)
	signed the PHRD (2011) kcal labelling pledge;<br>4. How common it was for chains not to provide instore kcal info but have info available on their websites		Verified by contacting companies, reviewing websites and conducting some physical audits		their websites or were able to provide this info on request.	
**USA (n=4)**						
Beckelman et al (2020)[39]<br><br>Choose Healthy Now<br>*Low (-)*	To describe the efforts of the Hawaiʻi State Department of Health to scale up the Choose Healthy Now programme through partnerships with 2 convenience store chains at a statewide level.	Cross-sectional<br><br>(2015)	Adults > 18 years living in Hawaii (n=162)<br><br>Exit store survey and Hawaiʻi Behavioral Risk Factor Surveillance System (statewide random digit–dial telephone survey).	Awareness of the programme and influence: Exit surveys with convenience store customers >18 years old.	Store survey exit: Convenience and price were the most influencing factors, followed by health and nutrition. Nearly half of Ps (n = 162; 46.3%) recalled seeing a programme's sign in the store when shown an example. Sign recall did not differ by age, gender, and ethnicity. Among those who recalled seeing a sign (n=75), 13.3% said that it influenced their purchases.<br>Telephone survey: one third (34.8%) said having seen or heard of a Choose Healthy Now campaign advertisement, especially native Hawaiian and other Pacific Islanders, who had a higher recall rate (43.6%)	Affiliations: University, Hawaiʻi State Department of Health;<br>Funding: Hawaiʻi State Department of Health, and US Centers for Disease Control and Prevention<br>CI: Declared having none.
Ng et al (2014)[56]<br><br>*HWC*<br><br>*Moderate (+)*	To evaluate the HWC companies' collective change in total calorie sales between 2007 and 2012 and to identify which food and beverage categories were major sources of the reductions or increases in calories sold.	Longitudinal (pre-post)<br><br>(2007 and 2012)	Calories sold from consumer packaged goods (CPG) sales data from mass merchandisers and convenience stores.<br><br>Data from Nielsen Scantrak, Nielsen Homescan from over 60,000 households, Nutrition Fact Label	Calories in food and beverages sold	Over the five years, total calories sold by the HWC companies fell from 60.4 trillion in 2007 to 54 trillion in 2012 (i.e., reduction of 6.4 trillion calories, or -10.6%).<br>Consumer packaged goods caloric sales (all brands) decreased by average by 78 kcal/capita/day from P brands, and by 11 kcal/capita/day from NP brands (adjusted).<br>Reductions from HWC brands came primarily from sweets and snacks (21 kcal); grain products (17 kcal) such as ready-to-eat cereal and granolas; fats and oils, sauces and condiments (15 kcal); beverages (14 kcal), particularly	Affiliations: University;<br>Funding: Robert Wood Johnson Foundation and Carolina Population Center;<br>CI: NIH, prior research funding from Nestle, gift from Kraft and Gerber Foods to co-fund a national dietary survey

Lead author (year) *PPP; study quality*	Study aim	Study design (Data collection dates)	Sample and data sources	Outcomes assessed	Results	Reported affiliations, funding, and competing interests (CI)
			from Gladson, Mintel GNPD, Datamonitor PLA		carbonated soft drinks (7 kcal); and shelf-stable fruit & vegetables drinks/juices (5 kcal).	
Ng & Popkin (2014)[55]<br><br>*HWC*<br><br>*Moderate (+)*	To assess the total CPG calories purchased per capita per day by households. To compare HWC name-brand products with non-HWC name-brand products and private label products. Using a counterfactual based on pre-pledge trends, see if the HWC contributed to a reduction in calories purchased	Repeat cross-sectional (pre-post)<br><br>(2000–2012)	CPG purchase data (food and beverages) from 61,126 households.<br><br>Nutrition info from Nielsen Homescan data, Nutrition Fact Label data from Gladson, Mintel GNPD, Datamonitor PLA	Calories purchased per capita per day by households	Unadjusted annualised caloric sales of CPG declined faster in the post pledge (2008-12) period than pre-pledge (2000-07).<br>Private labels saw sig declines post-pledge. HWC products saw the greatest absolute and relative declines in calories. The rate was statistically steeper post-pledge.<br>Non-HWC calorie declines across private label products slowed post-pledge, not sig.<br>A sig (p<0.001) reduction in CPG calories between 2007-12 of -206kcal/capita/day, -96kcal from HWC products, -63kcal from non-HWC and -47 from private labels. Post-pledge reductions were greater than pre-pledge trends (counterfactual).	Affiliations: University;<br>Funding: Robert Wood Johnson Foundation and Carolina Population Center;<br>CI: Prior research funding by Nestle's Water USA, gift from Kraft and Gerber Foods to co-fund a national dietary survey
Rajbhandari-Thapa et al (2017)[40]<br><br>*Strong4Life School Nutrition Programme*<br><br>*Low (-)*	To assess the effect of the Strong4Life SNP on (1) participant knowledge of evidence-based strategies for improving school cafeteria and student school meal practices and self-confidence in their ability to make changes, (2) the school cafeteria environment, and (3) National School Lunch Program participation.	Repeat cross-sectional (pre-post training a Strong4Life SNP 90-minute training session)<br><br>(2015)	School meal participation one month after the training: 80 Strong4Life schools, 40 of which took part in the training<br><br>Changes to the cafeteria environment: 842 managers and staff (325 at 3-month follow-up).<br><br>Questionnaires	Participation to the National School Lunch programme;	Participation in school lunch in the month following training did not change compared with a year before (trained schools: change= –0.2%, P=0.30; untrained schools: change= –0.5%, P=0.36).<br><br>Self-reported changes to the cafeteria environment: multiple results – copy-pasted below:<br>'For use of visibility strategies, the proportion of participants who reported having healthy options available in ≥2 locations on each service line increased from 84% to 96% (P < .001) and placing healthy foods in the first spots on the line increased from 55% to 67% (P < .001). For the convenience strategy, the	Affiliations: University, Child Wellness- Children's Healthcare of Atlanta<br>Funding: Strong4Life Program at Children's Healthcare of Atlanta.<br>CI: Authors employed by Children's Healthcare of Atlanta's Strong4Life program

Lead author (year) *PPP; study quality*	Study aim	Study design (Data collection dates)	Sample and data sources	Outcomes assessed	Results	Reported affiliations, funding, and competing interests (CI)
					proportion of participants reporting that they placed plain milk in front of flavored milk, juice, and sports drinks also increased significantly from 63% to 84% (P < .001). For strategies related to taste perception, the proportion of participants who reported using signs, posters, or decals showing the benefits of healthy eating increased from 77% to 85% (P ¼ .01). The proportion of participants who reported having _2 different colors of fruit and vegetables available daily increased from 92% to 98% (P < .001). In the sell focus area, participants using signage and/or floor decals to direct students toward service areas increased from 58% to 69% (P ¼ .01). We also found significant increases in the proportion of participants branding healthy items with stickers (from 25% to 38%; P < .001) and writing daily options on menu boards (from 69% to 77%; P ¼ .02). In the price focus area, we found significant increases in participant use of bundled pricing for healthy items to encourage sales (from 17% to 27%; P < .001) and pricing less healthy items higher than healthy items (from 18% to 32%; P < .001).'	

AUS: Australia; AWASH: the Australian Division of World Action on Salt and Health; CI: Competing interests; CPG: Consumer packaged goods; FHD: Food and Health Dialogue; HWC: Healthy Weight Commitment; Info: information; Na: Sodium; NHMRC: National Health and Medical Research Council; NIHR: National Institute for Health and Care Research; NP: Non-Participants; NR: Not reported; NZ: New Zealand; NZHF: New Zealand Heart Foundation; P: Participants; PAHO: Pan-American Health Organization; PHRD: Public Health Responsibility Deal; Sig: significant; SNP: School Nutrition Program; TFA: Trans-fatty acids.

The reference numbers are the same as those employed in the main manuscript.

References

34. Levi R, Probst Y, Crino M, Dunford EK. Evaluation of Australian soup manufacturer compliance with national sodium reduction targets. *Nutrition & Dietetics*. 2018;75(2):200-205. doi:10.1111/1747-0080.12392

36. Jones A, Magnusson R, Swinburn B, et al. Designing a Healthy Food Partnership: lessons from the Australian Food and Health Dialogue. *BMC Public Health*. 2016;16(1)doi:10.1186/s12889-016-3302-8

37. Dunford EK, Eyles H, Mhurchu CN, Webster JL, Neal BC. Changes in the sodium content of bread in Australia and New Zealand between 2007 and 2010: implications for policy. *Med J Aust*. 2011;195(6):346-9. doi:10.5694/mja11.10673

39. Beckelman T, Sinclair-White BM, McGurk MD, et al. Encouraging Adults to Choose Healthy Now: A Hawai'i Convenience Store Intervention. *Journal of Nutrition Education and Behavior*. 2020;52(3):330-334. doi:10.1016/j.jneb.2019.11.016

40. Rajbhandari-Thapa J, Bennett A, Keong F, Palmer W, Hardy T, Welsh J. Effect of the Strong4Life School Nutrition Program on Cafeterias and on Manager and Staff Member Knowledge and Practice, Georgia, 2015. *Public Health Reports*. 2017;132(2 suppl):48S-56S. doi:10.1177/0033354917723332

41. Trevena H, Neal B, Dunford E, Wu J. An evaluation of the effects of the Australian Food and Health Dialogue targets on the sodium content of bread, breakfast cereals and processed meats. *Nutrients*. 2014;6(9)doi:10.3390/nu6093802

42. Robinson E, Burton S, Gough T, Jones A, Haynes A. Point of choice kilocalorie labelling in the UK eating out of home sector: a descriptive study of major chain. *BMC Public Health*. 2019;19(1)doi:10.1186/s12889-019-7017-5

43. Knai C, James L, Petticrew M, Eastmure E, Durand M, Mays N. An evaluation of a public-private partnership to reduce artificial trans fatty acids in England, 2011-16. *European Journal of Public Health*. 2017;27(4)doi:10.1093/eurpub/ckx002

45. Christoforou AK, Dunford EK, Neal BC. Changes in the sodium content of Australian ready meals between 2008 and 2011. *Asia Pac J Clin Nutr*. 2013;22(1):138-43. doi:10.6133/apjcn.2013.22.1.10

46. Sparks E, Farrand C, Santos JA, et al. Sodium levels of processed meat in australia: Supermarket survey data from 2010 to 2017. *Nutrients*. 2018 2018;10(11):1686. doi:http://dx.doi.org/10.3390/nu10111686

47. Trevena H, Dunford E, Neal B, Webster J. The Australian Food and Health Dialogue – the implications of the sodium recommendation for pasta sauces. *Public Health Nutrition*. 2013;17(7)doi:10.1017/S1368980013001791

48. Elliott T, Trevena H, Sacks G, et al. A systematic interim assessment of the Australian Government's Food and Health Dialogue. *The Medical Journal of Australia*. 2014;200(2)doi:10.5694/mja13.11240

49. Lindberg R, Nichols T, Yam C. The healthy eating agenda in Australia. Is salt a priority for manufacturers? *Nutrients*. 2017 2017;9(8):881. doi:http://dx.doi.org/10.3390/nu9080881

51. Hutchinson J, Rippin HL, Jewell J, Breda JJ, Cade JE. Comparison of high and low trans-fatty acid consumers: analyses of UK National Diet and Nutrition Surveys before and after product reformulation. *Public Health Nutr*. 2018;21(3):465-479. doi:10.1017/S1368980017002877

52. Knai C, Petticrew M, Durand M, et al. Has a public–private partnership resulted in action on healthier diets in England? An analysis of the Public Health Responsibility Deal food pledges *Food Policy*. 2015;54:1-10.

55. Ng SW, Popkin BM. The healthy weight commitment foundation pledge: calories purchased by U.S. households with children, 2000-2012. *American journal of preventive medicine*. Oct 2014;47(4):520-30. doi:10.1016/j.amepre.2014.05.030

56. Ng SW, Slining MM, Popkin BM. The healthy weight commitment foundation pledge: calories sold from U.S. consumer packaged goods, 2007-2012. *Am J Prev Med*. Oct 2014;47(4):508-19. doi:10.1016/j.amepre.2014.05.029
